# Compatibility and combination of world *W*-boson mass measurements

**DOI:** 10.1140/epjc/s10052-024-12532-z

**Published:** 2024-05-02

**Authors:** LHC-TeV MW Working Group, S. Amoroso, N. Andari, W. Barter, J. Bendavid, M. Boonekamp, S. Farry, M. Grünewald, C. Hays, R. Hunter, J. Kretzschmar, O. Lupton, M. Pili, M. Ramos Pernas, B. Tuchming, M. Vesterinen, A. Vicini, C. Wang, M. Xu

**Affiliations:** 1 DESY, Hamburg, Germany; 2 CEA/IRFU, Gif-sur-Yvette, France; 3 University of Edinburgh, Edinburgh, UK; 4 Massachusetts Institute of Technology, Cambridge, MA USA; 5 University of Liverpool, Liverpool, UK; 6 University College Dublin, Dublin, Ireland; 7 University of Oxford, Oxford, UK; 8 University of Warwick, Warwick, UK; 9 University of Milan, Milan, Italy; 10 Johannes Gutenberg University, Mainz, Germany

## Abstract

The compatibility of *W*-boson mass measurements performed by the ATLAS, LHCb, CDF, and D0 experiments is studied using a coherent framework with theory uncertainty correlations. The measurements are combined using a number of recent sets of parton distribution functions (PDF), and are further combined with the average value of measurements from the Large Electron–Positron collider. The considered PDF sets generally have a low compatibility with a suite of global rapidity-sensitive Drell–Yan measurements. The most compatible set is CT18 due to its larger uncertainties. A combination of all $m_W$ measurements yields a value of $m_W = 80{,}394.6 \pm 11.5$ MeV with the CT18 set, but has a probability of compatibility of 0.5% and is therefore disfavoured. Combinations are performed removing each measurement individually, and a 91% probability of compatibility is obtained when the CDF measurement is removed. The corresponding value of the *W* boson mass is $80{,}369.2 \pm 13.3$ MeV, which differs by $3.6\sigma$ from the CDF value determined using the same PDF set.

## Introduction

The *W*-boson mass $(m_W)$ is a fundamental parameter of the Standard Model of particle physics (SM). Its value can be predicted by the SM and its measurement provides a sensitive test of the model’s consistency, offering a window to potential new processes. An active program of measurements at the Tevatron and Large Hadron Collider (LHC) continues to improve the experimental precision of $m_W,$ which is approaching the uncertainty on the SM prediction. Previous measurements from the Large Electron Positron collider (LEP) together have a precision comparable to the individual hadron-collider measurements. A combination of the Tevatron, LHC, and LEP measurements can thus improve the precision on $m_W$ and quantify the compatibility of the measurements. Such a compatibility study is particularly motivated in light of the discrepancy between the most recent measurement [1] from the CDF experiment at the Tevatron and previous measurements [2–5] from the D0 experiment at the Tevatron, and the LHCb and ATLAS experiments at the LHC.

At hadron colliders, measurements of $m_W$ exploit the kinematic peaks of distributions observed in leptonic *W*-boson decays. These final-state distributions carry information about the decaying particle mass, but also depend on other *W*-boson degrees of freedom such as the *W*-boson rapidity, transverse momentum, and polarization. Predictions of these distributions are obtained using Monte Carlo (MC) event generators with input parton distribution functions (PDF). Past measurements have used different generators and PDF sets, so prior to combining the measurements a coherent treatment is required to compare measurements and obtain uncertainty correlations. Where appropriate, small adjustments are thus applied to the measured values or uncertainties. These adjustments are estimated using a fast detector simulation developed for this purpose, or using the simulation from the experimental measurement.

The presentation of the combination begins with an overview of the individual measurements in Sect. 2, followed by a description of the methods in Sect. 3. The theoretical treatment of the *W*-boson production and decay is provided in Sect. 4, along with uncertainties, correlations, and any adjustments to the measurements. The results of the combination are presented in Sect. 5.2, and conclusions are given in Sect. 6.

## Overview of the measurements

The combination uses the latest measurements from D0 and CDF at the Tevatron and ATLAS and LHCb at the LHC. An $m_W$ measurement with the CMS detector is in progress, following the measurement of differential *W*-boson cross sections [6]. Prior measurements from the Tevatron and the CERN Super Proton Synchrotron are not included as they are expected to have negligible impact. The hadron-collider measurements are combined with the result from the Large Electron Positron collider (LEP) [7], $m_W = 80.376 \pm 0.033$ GeV.^1^

The kinematic observables used in $m_W$ measurements at hadron colliders are the transverse momentum^2^$(p^{\ell }_{{\text {T}}})$ and rapidity of the charged lepton from the *W*-boson decay, and the transverse momentum of the particles recoiling against the *W* boson $(u_{{\textrm{T}}}),$ whose transverse momentum is denoted $p_{{{\text {T}}}}^{W}$. The quantity $u_{{\textrm{T}}},$ referred to as the recoil, is measured by vectorially summing the momentum of all measured particles, except for the charged lepton. The neutrino momentum is inferred from the net momentum imbalance, $\textbf{p}^{\nu }_{{\textrm{T}}} \equiv -(\textbf{p}^{\ell }_{{\textrm{T}}} + \textbf{u}_{{\textrm{T}}}).$ For experiments with sufficiently good recoil resolution the most sensitive kinematic quantity is the transverse mass, $m_{{\text {T}}} = \sqrt{2p_{{\text {T}}}^{\ell } p_{{\text {T}}}^\nu (1-\cos \varDelta \phi )},$ where $\varDelta \phi$ is the angle between the charged lepton and the neutrino in the transverse plane. When the recoil resolution is an order of magnitude larger than the charged-lepton resolution, the most sensitive kinematic quantity is $p^{\ell }_{{\text {T}}}.$

The CDF Collaboration measured $m_W$ [1] using Run 2 data collected between 2003 and 2011 at the Tevatron collider, corresponding to 8.8 fb$^{-1}$ of integrated luminosity from proton-antiproton $(p\bar{p})$ collisions at a center-of-mass energy of $\sqrt{s}=1.96$ TeV. The mass was obtained from template fits to the reconstructed distributions of $p^{\ell }_{{\textrm{T}}},$ $m_{{{\text {T}}}}$, and $p^{\nu }_{\mathrm T}$ in the electron and muon decay channels, yielding $m_W=80{,}433.5\pm 6.4~(\text {stat.}) \pm 6.9~(\text {sys.})$ MeV, or $80{,}433.5\pm 9.4$ MeV. The quoted value of $m_W$ corresponds to the NNPDF3.1 PDF set [8], with the PDF uncertainty estimated using the largest 25 symmetric eigenvectors constructed through a principal-component analysis from the full replica set. The direct fit for $m_W$ to the data used events from a version of the ResBos [9] generator referred to as ResBos-C in this paper. The generation used the CTEQ6M PDF set [10] and was tuned to fit the observed spectrum of *Z*-boson transverse momentum. The uncertainty on the *W*-boson transverse momentum $p_{{\text {T}}}^{W}$ was determined using DYqT [11, 12], with a constraint from the observed recoil distribution in *W*-boson events. The adjustment of the model to the NNPDF3.1 PDF set included an effective update of the modelling of the leptonic angular distributions, as discussed in Sect. 4.3.

The D0 Collaboration performed two measurements of $m_W$ in Run 2 of the Tevatron collider. The first used data taken between 2002 and 2006, corresponding to an integrated luminosity of 1.1 fb$^{-1}$ [2], and the second used 2006–2008 data corresponding to an integrated luminosity of 4.3 fb$^{-1}$ [3]. The analysis produced template fits for $m_W$ using the $p^{\ell }_{{\text {T}}},$ $m_{{{\text {T}}}}$, and $p^{\nu }_{{\text {T}}}$ kinematic distributions in the electron decay channel. The initial 1.1 fb$^{-1}$ measurement combined the results from these three distributions, while the 4.3 fb$^{-1}$ measurement removed the $p^{\nu }_{{\text {T}}}$ result due to its small weight in the combination. The overall combined result of all measurements is $m_W = 80{,}375\pm 13~(\text {stat.})\pm 22~(\text {sys.})$ MeV, or $80{,}375\pm 23$ MeV. This value was determined using the CTEQ6.1 [13] (CTEQ6.6 [14]) PDF set for the measurement using 1.1 fb$^{-1}$ (4.3 fb$^{-1}$). The uncertainties were evaluated using Pythia6 [15] and the CTEQ6.1 PDF Hessian eigenvectors scaled to reduce the nominal 90% CL coverage to 68% CL. The $p_{{\text {T}}}^{W}$ modelling used a version of the ResBos [16, 17] generator referred to here as ResBos-CP.

The $m_W$ measurement performed by the ATLAS Collaboration used $\sqrt{s}=7$ TeV proton–proton collision data corresponding to 4.6 fb$^{-1}$ of integrated luminosity collected in 2011 during Run 1 of the LHC collider. ATLAS performed template fits to the $p^{\ell }_{{\text {T}}}$ and $m_{{{\text {T}}}}$ distributions in the electron and muon channels separately for $W^+$ and $W^-$ events, since in proton–proton (*pp*) collisions the final-state distributions are different for these processes. The fits were further subdivided into three (four) pseudorapidity ranges in the electron (muon) channel, yielding a total of 28 measurements. The combination of these measurements yields $m_W=80{,}370\pm 7~(\text {stat.})\pm 18~(\text {sys.}),$ or $80{,}370\pm 19$ MeV. The parton distribution functions were modelled with the NNLO CT10 PDF set [18], with the Hessian uncertainties scaled to 68% CL. The $p_{{\text {T}}}^{W}$ modelling relied on the parton shower Monte Carlo (MC) Pythia8 [19] tuned to match the $p_{{\text {T}}}^{Z}$ distribution observed in data. The impact of the PDF uncertainties on the $m_W$ measurement was reduced by a simultaneous fit in different lepton pseudorapidity regions. The PDFs affect both the $p_{{\text {T}}}^{W}$ and $p_{{\text {T}}}^{Z}$ distributions, and to preserve the agreement with the $p_{{\text {T}}}^{Z}$ data distribution only the relative variation between the $p_{{\text {T}}}^{W}$ and $p_{{\text {T}}}^{Z}$ distributions was propagated in the uncertainty estimate. Generated events were reweighted according to the calculation of the leptonic angular distributions in DYNNLO [20, 21].

The LHCb Collaboration performed a measurement of $m_W$ using Run 2 *pp* LHC collision data collected in 2016 at $\sqrt{s}=13$ TeV, corresponding to 1.7 fb$^{-1}$ of integrated luminosity. The measurement used the $q/p^{\ell }_{{\text {T}}}$ distribution in the muon decay channels, where *q* is the muon charge, giving a result of $m_W = 80{,}354 \pm 23~(\text {stat.}) \pm 10~(\text {exp.}) \pm 17~(\text {th.})\pm 9~(\text {PDF})$ MeV, or $80{,}354\pm 32$ MeV. The LHCb central value of $m_W$ and its uncertainty correspond to an unweighted average of results using the NNPDF3.1, MSHT2020 [22] and CT18 [23] PDF sets, all at next-to-leading order in the strong coupling and with 68% CL coverage. The $p_{{{\text {T}}}}^{W}$ distribution was modelled with Powheg [24–26] interfaced to Pythia8, with a correction at high boson $p_{{\text {T}}}$ derived from the observed *Z*-boson $p_{{\text {T}}}$ distribution. The leptonic angular distributions were modelled with exact ${\mathcal {O}}(\alpha _{{\text {S}}}^2)$ predictions from DYTurbo [27] and modified by scaling one of the leptonic angular coefficients when fitting the data.

The event requirements and fit ranges used in the measurements are summarized in Table 1. CDF and D0 used similar analysis configurations, while at ATLAS the looser recoil requirement and wider $m_{{{\text {T}}}}$ fit range were a consequence of the lower recoil resolution. The LHCb measurement was inclusive in recoil, with only a loose requirement on the momentum of the muon. The ATLAS, CDF, and D0 measurements fit $m_W$ only, while LHCb performed a simultaneous fit for $m_W$ and the relative fraction of $W^+$- to $W^-$-boson decays, the hadronic background fraction, $\alpha _{{\text {S}}}$ in *W*-boson events, $\alpha _{{\text {S}}}$ in *Z*-boson events, the intrinsic transverse momentum distribution of partons inside the proton, and the $A_3$ leptonic angular coefficient (see Sect. 4.3).

**Table 1:** Event requirements and fit ranges for CDF, D0, ATLAS, and LHCb

Experiment	Event requirements	Fit ranges
CDF	$30<p_{{{\text {T}}}}^{\ell }<55$ GeV	$32<p_{{{\text {T}}}}^{\ell }<48$ GeV
	$|\eta _{\ell }|<1$	$32<p_{{{\text {T}}}}^{\nu }<48$ GeV
	$30<p_{{{\text {T}}}}^{\nu }<55$ GeV	$60<m_{{{\text {T}}}}<100$ GeV
	$65<m_{{{\text {T}}}}<90$ GeV	
	$u_{{{\text {T}}}}<15$ GeV	
D0	$p_{{{\text {T}}}}^e >25$ GeV	$32<p_{{{\text {T}}}}^e<48$ GeV
	$|\eta _{\ell }|<1.05$	$65<m_{{{\text {T}}}}<90$ GeV
	$p_{{{\text {T}}}}^{\nu }>25$ GeV	
	$m_{{{\text {T}}}}>50$ GeV	
	$u_{{{\text {T}}}}<15$ GeV	
ATLAS	$p_{{{\text {T}}}}^{\ell }>30$ GeV	$32<p_{{{\text {T}}}}^{\ell }<45$ GeV
	$|\eta _{\ell }|<2.4$	$66<m_{{{\text {T}}}}<99$ GeV
	$p_{{{\text {T}}}}^{\nu }>30$ GeV	
	$m_{{{\text {T}}}}>60$ GeV	
	$u_{{{\text {T}}}}<30$ GeV	
LHCb	$p_{{{\text {T}}}}^\mu > 24$ GeV	$28< p_{{{\text {T}}}}^\mu < 52$ GeV
	$$2.2< \eta _\mu < 4.4$$	

## Methods

The combination consists of three steps. First, the results are adjusted to a common model to allow a consistent comparison of central values and evaluation of uncertainty correlations. This reference model includes the description of the *W*-boson production, the Breit–Wigner lineshape, and the *W*-boson polarization, and is described in Sect. 4. Second, the correlation of uncertainties between the experiments is evaluated. The different center-of-mass energies, initial states, and lepton pseudorapidity coverage make the correlation non-trivial. Finally, the results are combined for representative PDF sets, with the compatibility of the measurements determined for each set. In addition, other *W* and *Z* boson measurements at the Tevatron and LHC are compared to predictions using these PDF sets, in order to study the reliability of the PDF predictions and uncertainties for the $m_W$ measurement.

The first step of adjusting each result to a different theoretical model requires an emulation of the measurement process, which consists of Monte Carlo event generation (see Sect. 3.1), detector simulation (see Sect. 3.2), event selection, and a kinematic fit for $m_W.$ The Monte Carlo samples are produced using a reference value $m_W^{{{\text {ref}}}}$ for the *W*-boson mass and width $({\Gamma }_W),$ and different values of $m_W$ are obtained by reweighting events according to a Breit–Wigner distribution,

1 $$\begin{aligned} w(m,m_W,m_W^{{\text {ref}}}) = \frac{(m^2 - {m_W^{{\text {ref}}}}^2)^2 + m^4 {\Gamma }_W^2 / {m_W^{{\text {ref}}}}^2}{(m^2 - m_W^2)^2 + m^4 {\Gamma }_W^2 / m_W^2}, \end{aligned}$$

using the final-state invariant mass *m*. This parameterization uses the running width scheme in accordance with the published measurement procedures. The mass reweighting procedure has been checked to give the correct target mass value within a statistical uncertainty of $\approx 0.2$ MeV.

The detector simulations used in the original ATLAS, CDF, and D0 measurements are simplified so that large event samples can be simulated for a variety of PDF sets (see Sect. 3.2). These simulations do not have the complexity required for a mass measurement in data but are sufficient for estimating the impact of small theoretical modifications on the measurement. For the LHCb measurement no simplification is necessary and the same detector simulation is used as in the original measurement.

The shift in the value of $m_W$ resulting from a change in the generator model is estimated by producing template distributions using a given experiment’s model, and the same kinematic distributions for an alternate model (the “pseudo-data”). The shift is determined by minimizing the negative log-likelihood between the pseudo-data and the template distributions. In the following we quote the impact $\delta m_W$ of each theoretical shift on a measurement, i.e. the change in the measured $m_W$ value for a given change in the theoretical model.

A common set of uncertainties and correlations between experiments is obtained by evaluating $\delta m_W$ for a variety of PDF sets within the reference theoretical model. Summing the theoretical shifts gives a total $\delta m_W$ that we add to each experimental measurement to obtain the value to be used in the combination. For each PDF set the combination is performed using the method of the best linear unbiased estimator (BLUE) [28], including both theoretical and experimental uncertainties. The BLUE method is used by the individual experiments to combine results from different kinematic distributions, and the combination procedure is validated by reproducing each experiment’s published value. Results are presented for a combination of all experimental measurements, as well as for various measurement subsets.

### Monte Carlo event generation

The effects of modifying the *W*-boson production and decay model are studied using event samples for the $W \rightarrow \ell \nu$ process in *pp* collisions at $\sqrt{s}=7$ TeV and $\sqrt{s}=13$ TeV, and for $p\bar{p}$ collisions at $\sqrt{s}=1.96$ TeV. The event generators include those used by the original experiments, along with more recent versions with improved calculations. The PDF sets considered include those from the original measurements (CTEQ6M, CTEQ6.1, CTEQ6.6, CT10, NNPDF3.1, CT18, and MSHT2020), as well as the following sets at next-to-next-to-leading order (NNLO) in $\alpha _{{\text {S}}}$: NNPDF4.0 [29], ABMP16 [30], CT14 [31], and MMHT2014 [32].

For the ATLAS, CDF, and D0 experiments the $m_W$ shift associated with a particular NNLO PDF set is evaluated using the NNLO QCD calculation Wj-MiNNLO [33, 34] implemented in Powheg-Box-V2 [24–26]. The analysis is performed at the Les Houches event level [35] without interfacing to a parton shower, allowing for an efficient and fast processing. The addition of the parton shower has been confirmed to negligibly affect the shift associated with the PDF set.

The uncertainty associated with a given PDF set is evaluated using the next-to-leading order (NLO) QCD calculation W_ew-BMNNP [36] implemented in the Powheg-Box-V2. This calculation is used for efficiency reasons and the difference in the estimated uncertainty with respect to an NNLO calculation is expected to be negligible. For LHCb the $m_W$ shifts and uncertainties are evaluated using the same Powheg NLO calculation of *W*-boson production [37] as used in the original measurement.

The modelling of the *W*-boson polarization and resonance lineshape are studied using large ResBos samples corresponding to those from the Tevatron measurements: ResBos-C [16], used by CDF in their direct fit to the data [1], with an accuracy of NLO and approximate NNLL in QCD [38]; and ResBos-CP [17], used in the D0 $m_W$ measurement [3], with an accuracy of NNLO+NNLL in QCD. A third sample is generated using ResBos2 [38] with an accuracy of NLO+NNLL in QCD and including a full resummation of the coefficients describing the leptonic angular distributions (see Sect. 4.3). The difference between NLO and NNLO predictions of these coefficients is studied using the DYNNLO generator [20, 21], which has been confirmed to be consistent with other fixed-order calculations [39].

Electroweak corrections, primarily photon radiation in *W*-boson decay, have a large impact on the final-state distributions but are calculated accurately. The experiments factorize these corrections from PDF and QCD effects and we therefore do not include them in the sample generation.

### Detector simulations

This subsection provides brief descriptions of the parameterized simulations used to study the effects of model variations on the combination, and shows fit distributions comparing the simulations to those used in the experiments. The simulation of each detector is referred to as the “LHC-TeV MWWG” or “MWWG” simulation for that detector. The difference between the MWWG simulation and corresponding experimental simulation is ${{\mathcal {O}}}(1\%)$ for the fit distributions. A systematic uncertainty is taken to cover this difference and is applied by varying the lepton and recoil scales and resolutions by $\pm 5$%, which is approximately an order of magnitude larger than the experimental uncertainty. These uncertainties lead to ${{\mathcal {O}}}$(1 MeV) uncertainties on the impact of theoretical variations.

#### CDF response and resolution model

The CDF detector model consists of parameterizations of the electron and recoil momentum response and resolution. The muon momentum response and resolution are similar to those of electrons.

The electron fractional momentum resolution $\sigma _{p_{\tiny {\text {T}}}}/p_{{{\text {T}}}}= \sqrt{\kappa ^2 + S^2/p_{{{\text {T}}}}}$ is modelled using a sampling term of $S=12.6\%$ GeV$^{1/2}$ and a constant term of $\kappa =2\%.$ The constant term is larger than that used in the CDF simulation in order to correct for the lack of final-state radiation in the generated samples. The leakage of the shower beyond the electromagnetic calorimeter is parameterized in the same manner as for the CDF measurement, and the reduction in electron momentum is corrected with a scale factor applied to the electron momentum.

The recoil response is defined as the ratio $R(p_{{{\text {T}}}}^{W})$ of the measured recoil $u_T$ to the generated $p_{{{\text {T}}}}^{W},$ before accounting for effects of underlying event and additional proton-antiproton interactions (pileup). The CDF response is parameterized as

2 $$\begin{aligned} R(p_{{{\text {T}}}}^{W}) = r \log (a p_{{{\text {T}}}}^{W})/ \log (a p_{{{\text {T}}}}^{{\text {ref}}}), \end{aligned}$$

where $r=0.65,$ $a=6.7/$GeV, and $p_{{{\text {T}}}}^{{\text {ref}}} = 15$ GeV.

The jet-like sampling for the recoil resolution is

3 $$\begin{aligned} \sigma (p_{{{\text {T}}}}^{W}) = s \sqrt{p_{{{\text {T}}}}^{W}}, \end{aligned}$$

with $s=0.87$ GeV$^{1/2}$ and $p_{{{\text {T}}}}^{W}$ in GeV. The recoil azimuthal angular resolution $\sigma _{u_\phi }$ is parameterized as

4 $$\begin{aligned} \sigma _{u_\phi }(p_{{{\text {T}}}}^{W}) = \alpha - \beta \times p_{{{\text {T}}}}^{W}, \end{aligned}$$

where $\alpha =0.273$ rad and $\beta =0.016$ rad/GeV for $p_{{{\text {T}}}}^{W}<p_{{{\text {T}}}}^{{\text {ref}}},$ and $\alpha =0.143$ rad and $\beta =0.0044$ rad/GeV for $p_{{{\text {T}}}}^{W}\ge p_{{{\text {T}}}}^{{\text {ref}}}.$

The contribution of the underlying event to the measured recoil is represented by a randomly-oriented Gaussian distribution of width 6.2 GeV. Finally, the removal of lepton calorimeter towers from the recoil reconstruction is modelled by subtracting 660 MeV from the generated recoil along the direction of the decay lepton.

**Fig. 1:**
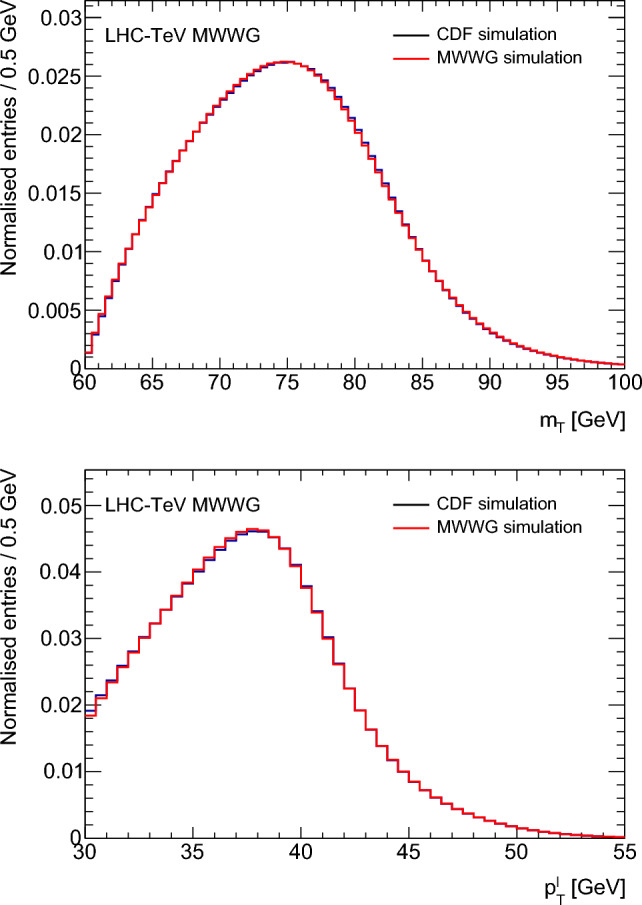
Comparisons between the CDF simulation [40] and the LHC-TeV MWWG simulation for the $m_{{{\text {T}}}}$ (top) and $p_{{{\text {T}}}}^{\ell }$ (bottom) distributions

The distributions obtained using the MWWG simulation are compared with those from the CDF simulation in Fig. 1. The agreement is at the percent level in the range of interest for the measurement. The systematic uncertainties on the MWWG simulation are estimated by varying the response and resolution by $\pm 5\%,$ calculating the $\delta m_W$ shifts for fourteen PDFs, and taking the maximum $|\delta m_W|.$ The resulting uncertainties are 1.0 MeV for the $m_{{{\text {T}}}}$ fit, 0.9 MeV for the $p_{{{\text {T}}}}^{\ell }$ fit, and 2.0 MeV for the $p_{{{\text {T}}}}^{\nu }$ fit.

#### D0 response and resolution model

The LHC-TeV MWWG simulation of the D0 detector includes a model of the efficiency of the electron reconstruction and selection along with the response and resolution of the recoil and the electron momentum. The simulation reproduces distributions from the D0 parameterized Monte Carlo simulation (PMCS) used for the final D0 measurement based on an integrated luminosity of 4.3 fb$^{-1}$ [3]. The prior measurement based on 1.1 fb$^{-1}$ [2] of integrated luminosity has a lower mean number of pileup events; the corresponding impact on the estimation of mass shifts is within the applied uncertainty.

The electron energy response is parameterised as:

5 $$\begin{aligned} E = \alpha (E_0-\bar{E_0}) + \beta + \bar{E_0}, \end{aligned}$$

where *E* is the calibrated electron energy, $\bar{E_0}=43$ GeV is a reference value corresponding to the electron energy in *Z*-boson events, and $\alpha$ and $\beta$ are luminosity-dependent energy scale and offset corrections, respectively. We take $\alpha =1.0164$ and $\beta =0.188$ GeV, the values determined in Ref. [3] for an instantaneous luminosity in the range (2–4) $\times 36\times 10^{30}$ ${\text {cm}}^{-2}{\text {s}}^{-1}$ corresponding to the largest fraction of the data. Implementing the instantaneous luminosity dependence gives results in agreement with the average response to within a percent.

The electron energy resolution $\sigma _E/E$ is simulated using the same functional form as for CDF, with a constant term of $\kappa =1.997\%$ [3] and a sampling term of

6 $$\begin{aligned} S= S_0 \exp \left[ S_1\left( \frac{1}{\sin \theta }-1\right) \right] + \frac{S_2\eta +S_3}{\sqrt{E}}, \end{aligned}$$

where $S_0=0.153$ GeV$^{1/2},$ $S_1=1.543,$ $S_2=-0.025$ GeV, $S_3=0.172$ GeV, and *E* is in GeV. The resulting fractional resolution is increased by 2% to account for the lack of generated final-state radiation and improve the agreement with the distributions from the D0 PMCS.

The electron reconstruction and identification efficiency is modeled by the following function determined using the data points in Fig. 25(b) of Ref. [3]:

7 $$\begin{aligned} \varepsilon ( p_{{{\text {T}}}}^{\ell })= 0.95 \left( 1- e^{-{0.074 p_{{{\text {T}}}}^{\ell }}}\right) , \end{aligned}$$

where $p_{{{\text {T}}}}^{\ell }$ is in GeV.

The recoil is modelled using a migration matrix to obtain a simulated $u_{{{\text {T}}}}$ value for a given generated $p_{{{\text {T}}}}^{W}$ [41]. In order to model the recoil energy in the electron cone that is not included in the recoil measurement, 150 MeV are subtracted from the recoil component parallel to the decay lepton [3].

**Fig. 2:**
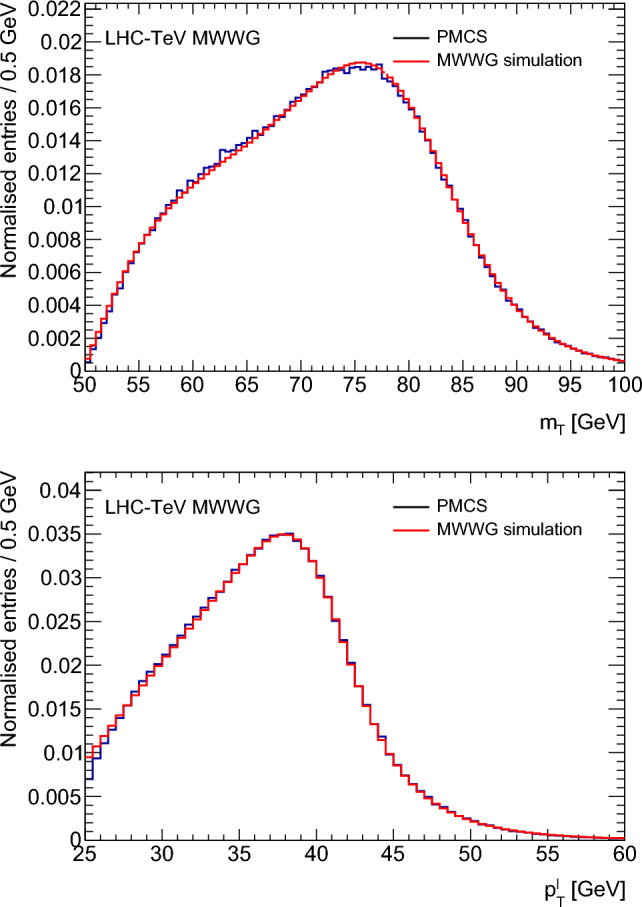
Comparisons of the D0 PMCS and the LHC-TeV MWWG simulation for the $m_{{{\text {T}}}}$ (top) and $p_{{{\text {T}}}}^{\ell }$ (bottom) distributions

Figure 2 shows the $p_{{{\text {T}}}}^{\ell }$ and $m_{{{\text {T}}}}$ distributions from the D0 PMCS and the LHC-TeV MWWG simulation after reweighting the events to match the $p_{{{\text {T}}}}^{W}$ distribution used for the D0 measurement. The distributions agree to within 2% in the range of interest for the $m_W$ extraction. The shifts in $m_W$ are studied for the eigenvectors of the CTEQ6.6 and CT10 PDFs, and the MWWG simulation and D0 PMCS agree within the statistical precision of $\approx 1$ MeV. Systematic uncertainties are determined by varying the scales and resolutions, and calculating the effect on $\delta m_W$ for fourteen PDF sets. The resulting uncertainties are 1.0 MeV on the $m_{{{\text {T}}}}$ fit, 1.0 MeV on the $p_{{{\text {T}}}}^{\ell }$ fits, and 2.2 MeV on the $p_{{{\text {T}}}}^{\nu }$ fit.

#### ATLAS response and resolution model

**Fig. 3:**
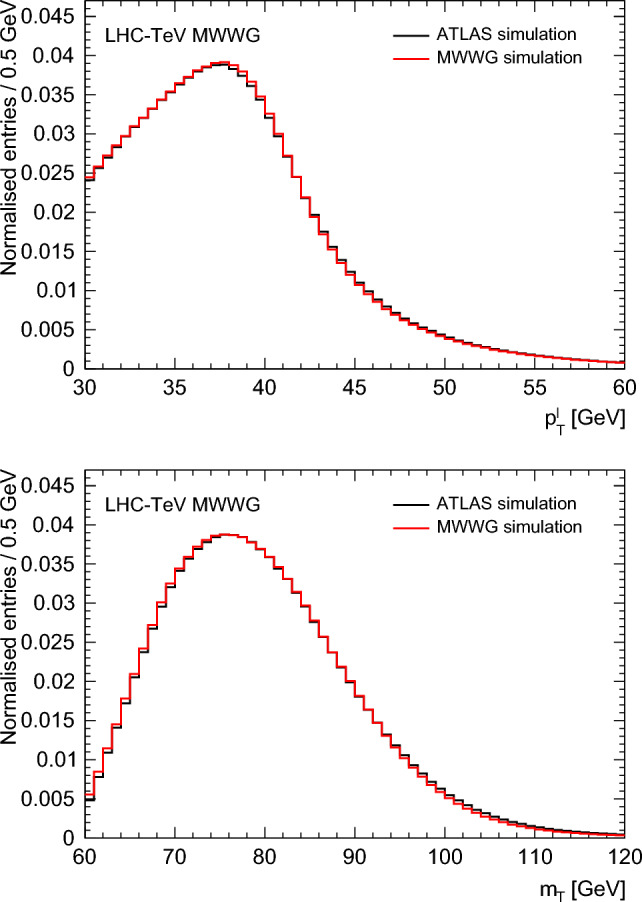
Comparison of the published and MWWG simulated $p_{{{\text {T}}}}^{\ell }$ (top) and $m_{{{\text {T}}}}$ (bottom) distributions for ATLAS

The ATLAS recoil response and resolution are parametrized using distributions [5] of the projections of these quantities along and perpendicular to the lepton direction, as a function of the *W*-boson transverse momentum. The parameterizations are calibrated using the full ATLAS simulation. The recoil resolution ranges from 12 to 16 GeV, depending primarily on the amount of pileup. The electron and muon resolutions are parameterized using the documented detector performance [42, 43]. The resulting $p_{{{\text {T}}}}^{\ell }$ and $m_{{{\text {T}}}}$ distributions are given in Fig. 3, which shows that the resolution is accurately modeled and that residual differences could be improved with lepton energy scale adjustments and do not significantly affect the results.

The accuracy of the LHC-TeV MWWG simulation in determining $m_W$ shifts is studied using PDF variations from the ATLAS measurement. With 28 measurement categories and 25 CT10 PDF eigensets, a statistically accurate comparison is made between the emulated measurement procedure and the results of the ATLAS measurement. A root-mean-square spread of 1.5 MeV is found between the published and emulated shifts in the various categories and eigensets. The differences dominantly reflect approximations in the Powheg-based reweighting procedure compared to the kinematic reweighting to NNLO-accurate distributions implemented in Ref. [5]. Systematic uncertainties are assessed by varying the response and resolution by $\pm 5\%,$ and are 1.1 MeV for the $p_{{{\text {T}}}}^{\ell }$ fit and 1.2 MeV for the $m_{{{\text {T}}}}$ fit.

## *W*-boson production and decay

The process of *W*-boson production and decay is similar in *pp* and $p\bar{p}$ collisions, with differences arising mainly in the parton distribution functions. Different PDF sets use different input data sets and procedures, and the correlation between sets cannot be readily calculated. Thus the combination is performed by adjusting the $m_W$ measurements to a common PDF set through the addition of a $\delta m_W^{{\text {PDF}}}$ specific to each experimental result. Events generated with the Wj-MiNNLO Monte Carlo are used to evaluate the corresponding PDF uncertainty and correlations. A separate shift $\delta m_W^{{\text {pol}}}$ is calculated to update the Resbos-C and Resbos-CP treatment of the *W* boson polarization to Resbos2. The line shape of the dilepton invariant mass is also studied, and adjustments are made for differences in the spectrum due to the CDF generator-level requirements $(\delta m_W^{{\text {gen}}})$ or to the assumed decay width in the measurements $(\delta m_W^{\Gamma }).$ Finally, correlations are estimated for uncertainties due to electroweak corrections such as final-state photon radiation.

### *W*-boson $p_{{{\text {T}}}}$ distribution

In the region relevant to the $m_W$ measurement, the $p_{{{\text {T}}}}^{W}$ distribution is described by a combination of perturbative fixed-order QCD, soft-gluon resummation, and non-perturbative effects. The Tevatron experiments use analytical resummation as implemented in ResBos-C and ResBos-CP, while ATLAS and LHCb use the Pythia8 parton shower interfaced to Powheg.

Non-perturbative effects influence the very low boson $p_{{{\text {T}}}}^{W}$ region, typically $p_{{{\text {T}}}}^{W}<5$ GeV, and are generally assumed to be universal between *W* and *Z* production (up to differences in $\sqrt{s}).$ In the absence of precise direct measurements of the *W*-boson $p_{{{\text {T}}}}$ distribution, all measurements use *Z*-boson data to constrain the non-perturbative parameters. The resulting model is then used for the *W*-boson $p_{{{\text {T}}}}$ distribution. The associated uncertainty accounts for the limited precision of the *Z*-boson data and for differences between the *Z*- and *W*-boson production mechanisms, in particular related to the different initial-state parton configurations.

**Fig. 4:**
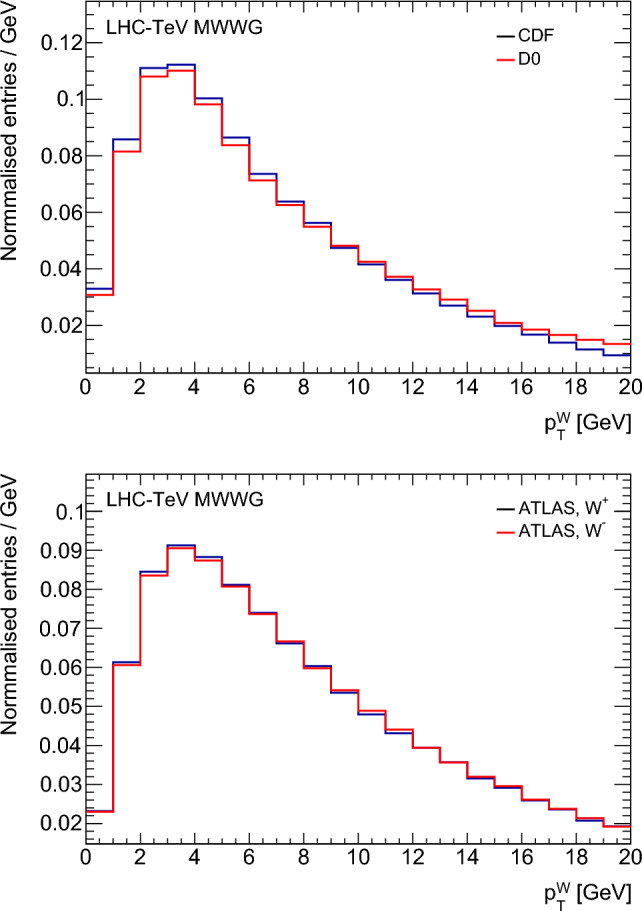
Distributions of generated $p_{{{\text {T}}}}^{W}$for $W^\pm$ candidate events in $p\bar{p}$ collisions at CDF and D0 (top), and for $W^+$ and $W^-$ events at ATLAS (bottom). The distributions represent the best-fit model resulting from the analysis of *W*- and *Z*-boson data in the respective experiments, and are shown after all event selection requirements

To describe the $p_{{{\text {T}}}}^{W}$ distribution, ATLAS and LHCb tune in situ the shower and non-perturbative parameters in Pythia (intrinsic $k_{{\text {T}}}$ and $\alpha _{{\text {S}}}),$ and LHCb adds an $\alpha _{{\text {S}}}$ tune in Powheg. The ATLAS tunes use the $p_{{{\text {T}}}}^{Z}$ distribution while the LHCb tunes use an angular distribution in $Z\rightarrow \mu \mu$ decays as well as the $q / p_{{{\text {T}}}}$ distribution used for the $m_W$ fit. CDF fits the non-perturbative resummation parameters $g_1,g_2$ in ResBos-C using the $p_{{{\text {T}}}}^{Z}$ distribution, and D0 uses the default values of these parameters in ResBos-CP. CDF additionally constrains the region above the peak with a fit for $\alpha _{{\text {S}}}.$ The resulting Tevatron and ATLAS $p_{{{\text {T}}}}^{W}$ distributions, after event selection and using the detector simulations described in Sect. 3.2, are shown in Fig. 4.

Theoretical uncertainties in the extrapolation from the $p_{{{\text {T}}}}^{Z}$ distribution to the $p_{{{\text {T}}}}^{W}$ distribution are considered by the ATLAS and CDF experiments, which use the observed *W*-boson $p_{{{\text {T}}}}$ distribution to validate (ATLAS) or further constrain (CDF) the associated uncertainty in situ. CDF (D0) quotes an uncertainty due to the *W*-boson $p_{{{\text {T}}}}$ modelling of 2.2 (2.4) MeV and ATLAS quotes 6.0 MeV. For LHCb an 11 MeV uncertainty is assessed using the envelope of fit results from Pythia8 (without Powheg), Powheg matched to Pythia8 or Herwig, and Herwig with its own matrix-element calculation. The different uncertainty values are due to differences in recoil requirements, the increased prevalence of heavy-flavour quarks in the initial state at the LHC, and different in situ constraints. Since the *W*-boson $p_{{{\text {T}}}}$ distributions are modelled with different generators or parameter values between the experiments, the corresponding uncertainties are taken to be uncorrelated.

### Parton distribution functions

#### Central values and uncertainty correlations

The $\delta m_W^{{\text {PDF}}}$ shift for each PDF set is evaluated for the Tevatron experiments and ATLAS using the precise NNLO Wj-MiNNLO calculation. The resulting shifts are compared to those from the NLO W_ew-BMNNP and Resbos calculations, and the differences are typically within the statistical precision of the comparison. For LHCb the PDF shift is determined with a direct fit to the data as in the original measurement.

All experimental measurements include an in situ constraint on $p_{{{\text {T}}}}^{Z}$ and/or $p_{{{\text {T}}}}^{W}$, as described in Sect. 4.2.1. We preserve these constraints by reweighting the relevant boson $p_{{{\text {T}}}}$ distribution for each PDF set to match that used in the measurement. For the Tevatron experiments $p_{{{\text {T}}}}^{W}$ is reweighted, while in the case of ATLAS $p_{{{\text {T}}}}^{Z}$ is reweighted since the lower recoil resolution does not provide a significant $p_{{{\text {T}}}}^{W}$ constraint from the data. For LHCb a constraint on $p_{{{\text {T}}}}^{W}$ is applied as part of the direct fit to the data for each PDF set.

For each PDF set $\delta m_W^{{\text {PDF}}}$ is evaluated using a common boson $p_{{{\text {T}}}}$ distribution across PDFs separately for each experiment. For the Tevatron experiments the *W* boson $p_{{{\text {T}}}}$ is reweighted to match that of the original measurement, due to the observed agreement between the measured recoil distribution and the model. In the case of ATLAS the *Z*-boson $p_{{{\text {T}}}}$ is reweighted to match the original measurement, since the lower recoil resolution does not provide a significant *W*-boson $p_{{{\text {T}}}}$ constraint from the data. For LHCb the PDF shift is determined with a direct fit to the data as in the original measurement, including constraints on $p_{{{\text {T}}}}^{W}$ and the most relevant polarization coefficient.

**Table 2:** Values of $\delta m_W^{{\text {PDF}}}$ in MeV for each PDF set using the $m_{{{\text {T}}}}$ fit distribution, determined using the Wj-MiNNLO calculation

PDF set	D0	CDF	ATLAS $W^+$	ATLAS $W^-$
CTEQ6	$-$ 14.6	0.0	–	–
CTEQ6.6	0.0	14.2	–	–
CT10	$-$ 0.5	14.3	0.0	0.0
CT14	$-$ 8.7	5.2	$-0.5$	$-7.6$
CT18	$-$ 7.5	6.5	13.4	$-5.5$
ABMP16	$-$ 17.9	$-$ 2.4	$-25.7$	$-7.9$
MMHT2014	$-$ 10.1	4.5	$-3.6$	9.1
MSHT20	$-$ 12.9	2.5	$-22.3$	4.2
NNPDF3.1	$-$ 1.0	13.1	$-14.6$	$-6.3$
NNPDF4.0	6.2	20.1	$-23.3$	4.3

In order to facilitate the evaluation of uncertainty correlations, Hessian eigenvector sets are used. The upper and lower uncertainties are taken to be

8 $$\begin{aligned} \sigma _{m_{W}^+}= & {} \left[ \sum _i \left( {\sigma _{m_{W}^i}}\right) ^2\right] ^{1/2} \text { if } \sigma _{m_{W}^i}>0\quad \text {and}\quad \nonumber \\ \sigma _{m_{W}^-}= & {} \left[ \sum _i \left( {\sigma _{m_{W}^i}}\right) ^2\right] ^{1/2} \text { if } \sigma _{m_{W}^i}<0, \end{aligned}$$

where *i* runs over the uncertainty sets. The uncertainties are symmetrized according to $\sigma _{m_{W}} = (\sigma _{m_{W}^+} + \sigma _{m_{W}^-})/2.$ For CTEQ PDF sets the translation from 90% CL to 68% CL assumes a gaussian distribution, i.e. a division by 1.645. The effect of each PDF eigenset is correlated across experiment or measurement category, and its contribution to the covariance between any two measurements $\alpha$ and $\beta$ is given by $C^i_{\alpha \beta } = \sigma _{m_{W\alpha }^i} \sigma _{m_{W\beta }^i}.$ Accounting for all eigensets of a given PDF, the total uncertainty covariance and the corresponding uncertainty correlation are calculated as

9 $$\begin{aligned} C^{{\text {PDF}}}_{\alpha \beta } = \sum _i C^i_{\alpha \beta },\quad \text {and}\quad \rho _{\alpha \beta } = \frac{\sum _i \sigma _{m_{W\alpha }^i} \sigma _{m_{W\beta }^i}}{\sigma _{m_{W\alpha }}\sigma _{m_{W\beta }}}. \end{aligned}$$

**Table 3:** Values of $\delta m_W^{{\text {PDF}}}$ in MeV for each PDF set using the $p_{{{\text {T}}}}^{\ell }$ (all experiments) or $p_{{{\text {T}}}}^{\nu }$ (CDF and D0) distribution, determined using the Wj-MiNNLO calculation

PDF set	D0 $p_{{{\text {T}}}}^{\ell }$	D0 $p_{{{\text {T}}}}^{\nu }$	CDF $p_{{{\text {T}}}}^{\ell }$	CDF $p_{{{\text {T}}}}^{\nu }$	ATLAS $W^+$	ATLAS $W^-$	LHCb
CTEQ6	$-$ 17.0	$-$ 17.7	0.0	0.0	–	–	–
CTEQ6.6	0.0	0.0	15.0	17.0	–	–	–
CT10	0.4	$-$ 1.3	16.0	16.3	0.0	0.0	–
CT14	$-$ 9.7	$-$ 10.6	5.8	6.8	$-1.2$	$-5.8$	1.1
CT18	$-$ 8.2	$-$ 9.3	7.2	7.7	12.1	$-2.3$	$-6.0$
ABMP16	$-$ 19.6	$-$ 21.5	$-$ 1.4	$-$ 2.4	$-22.5$	$-3.1$	7.7
MMHT2014	$-$ 10.4	$-$ 12.7	6.1	5.5	$-2.6$	9.9	$-10.8$
MSHT20	$-$ 13.7	$-$ 15.4	3.6	4.1	$-20.9$	4.5	$-2.0$
NNPDF3.1	$-$ 1.0	$-$ 1.2	14.0	15.1	$-14.1$	$-1.8$	6.0
NNPDF4.0	6.7	8.1	20.8	24.1	$-22.4$	6.9	8.3

**Table 4:** Uncertainty in MeV for each PDF set after combining the individual fit categories

PDF set	D0	CDF	ATLAS	LHCb
CTEQ6	–	14.1	–	–
CTEQ6.6	15.1	–	–	–
CT10	–	–	9.2	–
CT14	13.8	12.4	11.4	10.8
CT18	14.9	13.4	10.0	12.2
ABMP16	4.5	3.9	4.0	3.0
MMHT2014	8.8	7.7	8.8	8.0
MSHT20	9.4	8.5	7.8	6.8
NNPDF3.1	7.7	6.6	7.4	7.0
NNPDF4.0	8.6	7.7	5.3	4.1

**Fig. 5:**
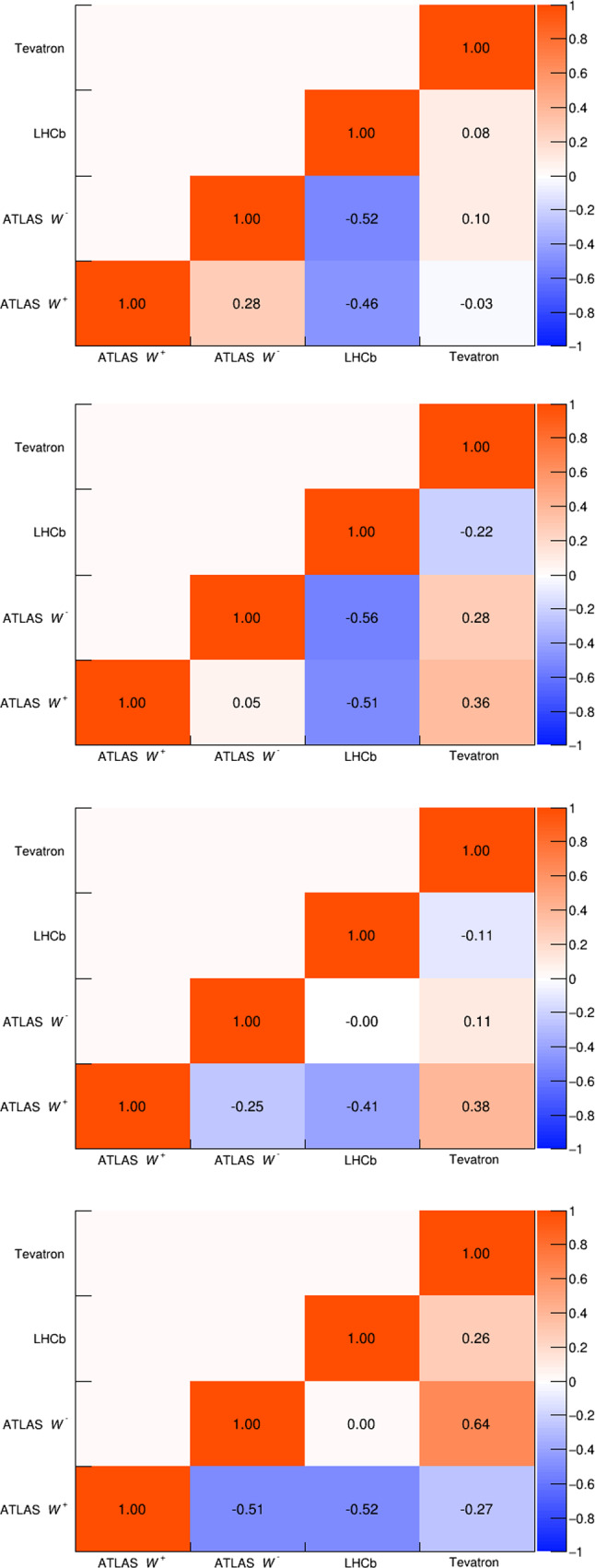
PDF uncertainty correlation matrices for the CT18, MSHT20, NNPDF4.0, and ABMP16 PDF sets, in order from top to bottom

Tables 2 and 3 show $\delta m_W^{{\text {PDF}}}$ for each PDF set and each experiment using distributions based on the transverse mass and the lepton or neutrino $p_T,$ respectively. For simplicity the ATLAS shifts are shown inclusively in lepton $\eta ,$ though separated by boson charge. The PDF uncertainties for each measurement are shown in Table 4, and the correlation matrices for the most recent PDF sets are shown in Fig. 5. Correlation matrices for older sets are provided in the Appendix.

#### *W*- and *Z*-boson production measurements

The various kinematic distributions and fiducial regions used to fit $m_W$ in the ATLAS and Tevatron experiments provides some sensitivity to PDF predictions. Other *W*- and *Z*-boson measurements from the LHC and the Tevatron provide more significant PDF constraints and are used in the determination of the PDF sets. This section compares the compatibility of these other measurements with the various PDF sets. Some sets have low compatibility and are not favoured for an $m_W$ combination.

The *W*-boson rapidity $(y_W)$ distribution affects the $m_W$ measurement through the $p_{{{\text {T}}}}^{\ell }$ distribution: more central *W* bosons can have more forward-decaying leptons within the detector acceptance, lowering the mean observed $p_{{{\text {T}}}}^{\ell }$. Measurements that probe PDF parameters describing $y_W$ include the *Z* boson rapidity $y_Z$ and the asymmetries in the rapidity distribution between positive and negative *W* bosons $(A_W),$ or similarly the positive and negative charged leptons from their decay $(A_\ell ).$ These measurements are considered in this compatibility study, and are shown in Table 5.

The comparison between data and predictions is performed with the xFitter [44] framework. A $\chi ^2$ measure is constructed including all experimental uncertainties and their correlations, as well as the PDF uncertainties. Theory predictions are calculated at NNLO in QCD and corrected to NLO electroweak predictions using multiplicative *k*-factors in each measurement bin. PDF uncertainties are computed at NLO in QCD using Applgrids [45] with calculations from MCFM-6.8 [46]. The results for various PDF sets are shown in Table 6.

Most of the Drell–Yan measurements have good $\chi ^2$ values for all PDFs. The most significant outlier is the D0 $W\rightarrow e \nu$ lepton asymmetry measurement, for which the CT18 set has the lowest $\chi ^2$ primarily due to its larger uncertainties. These larger uncertainties also reduce the correlated $\chi ^2,$ which represents the contribution from correlated uncertainties [47]. The correlated $\chi ^2$ reduces from 251 to 43 after including PDF uncertainties in the CT18 set; the corresponding reduction for the NNPDF3.1 set is 110 to 76. The overall probability of consistency of the combined datasets is 1.5% for the CT18 set, and is much lower for the other sets. Among the studied PDF sets CT18 is therefore considered to give the most accurate estimate of the 68% CL interval for combined *W*- and *Z*-boson measurements.

### *W*-boson polarization

The *W*-boson polarization affects the lepton decay angles, and in turn the transverse momentum of the leptons. A general expression for the fully differential distribution of the charged lepton is

10 $$\begin{aligned} \frac{d\sigma }{dp_{{{\text {T}}}}^{W}dy dm d\varOmega }= & {} \frac{d\sigma }{dp_{{{\text {T}}}}^{W}dy dm} [(1 + \cos ^2\theta ) \nonumber \\{} & {} + \frac{1}{2} A_0 (1-3\cos ^2\theta ) + A_1 \sin 2\theta \cos \phi \nonumber \\{} & {} + \frac{1}{2}A_2 \sin ^2\theta \cos 2\phi + A_3 \sin \theta \cos \phi \nonumber \\{} & {} + A_4 \cos \theta + A_5 \sin ^2\theta \sin 2\phi \nonumber \\{} & {} + A_6 \sin 2\theta \sin \phi + A_7 \sin \theta \sin \phi ], \end{aligned}$$

where the decay angles $\theta , \phi$ are expressed in the Collins–Soper (C-S) frame [54], and the $A_i$ coefficients depend on the $p_{{{\text {T}}}}$, rapidity, and invariant mass of the $\ell \nu$ system. The coefficients can be calculated perturbatively in $\alpha _{\textrm{S}},$ with $A_5,~A_6,$ and $A_7$ becoming non-zero only at NNLO in QCD. The $A_0$ term primarily reflects the relative fractions of the $q\bar{q}' \rightarrow W,$ $qg\rightarrow Wq,$ and higher-order subprocesses, and has a significant $p_{{{\text {T}}}}^{W}$ dependence while being nearly independent of boson rapidity. The $A_4$ term produces a forward-backward asymmetry, and is thus sensitive to the directions of the incoming quark and anti-quark in the dominant $q\bar{q}' \rightarrow W$ process. It depends on rapidity and on the PDF set used in the calculation, and decreases with increasing $p_{{{\text {T}}}}^{W}$.

**Table 5:** Drell–Yan measurements used for the PDF compatibility study

Exp.	Obs.	Decay	$\sqrt{s}$ (TeV)	Lum. (fb$^{-1}$)	Bins
CDF [48]	$A_W$	$e\nu$	1.96	1	13
CDF [49]	$y_Z$	*ee*	1.96	2.1	28
D0 [50]	$y_Z$	*ee*	1.96	0.4	28
D0 [51]	$A_\ell$	$\mu \nu$	1.96	7.3	12
D0 [52]	$A_\ell$	$e\nu$	1.96	9.7	13
ATLAS [53]	*Z*, *W*	$\ell \ell ,$ $\ell \nu$	7	4.7	61

**Table 6:** $\chi ^2$ per degree of freedom for the Tevatron *Z*-rapidity and *W*- and *l*-asymmetry measurements at $\sqrt{s}=1.96$ TeV, and the LHC *Z*-rapidity and *W* lepton-rapidity measurements at $\sqrt{s}=7$ TeV. The total $\chi ^2$ is the sum of those quoted for individual measurements along with a separate contribution for correlated uncertainties, where the latter is extracted using a nuisance parameter representation of the $\chi ^2$ [47]. The CT14 and CT18 PDF uncertainties correspond to 68% coverage, obtained by rescaling the eigenvectors by a factor of 1/1.645. The probability of obtaining a total $\chi ^2$ at least as high as that observed is labelled p$(\chi ^2,n)$

Measurement	NNPDF3.1	NNPDF4.0	MMHT14	MSHT20	CT14	CT18	ABMP16
CDF $y_Z$	24/28	28/28	30/28	32/28	29/28	27/28	31/28
CDF $A_W$	11/13	14/13	12/13	28/13	12/13	11/13	21/13
D0 $y_Z$	22/28	23/28	23/28	24/28	22/28	22/28	22/28
D0 $W \rightarrow e\nu$ $A_\ell$	22/13	23/13	52/13	42/13	21/13	19/13	26/13
D0 $W \rightarrow \mu \nu$ $A_\ell$	12/10	12/10	11/10	11/10	11/10	12/10	11/10
ATLAS peak CC $y_Z$	13/12	13/12	58/12	17/12	12/12	11/12	18/12
ATLAS $W^{-}$ $y_\ell$	12/11	12/11	33/11	16/11	13/11	10/11	14/11
ATLAS $W^{+}$ $y_\ell$	9/11	9/11	15/11	12/11	9/11	9/11	10/11
Correlated $\chi ^2$	75	62	210	88	81	41	83
Total $\chi ^2$/d.o.f.	200/126	196/126	444/126	270/126	210/126	162/126	236/126
p$(\chi ^2,n)$	0.003%	0.007%	$<10^{-10}$	$<10^{-10}$	$0.0004\%$	1.5%	$10^{-8}$

**Fig. 6:**
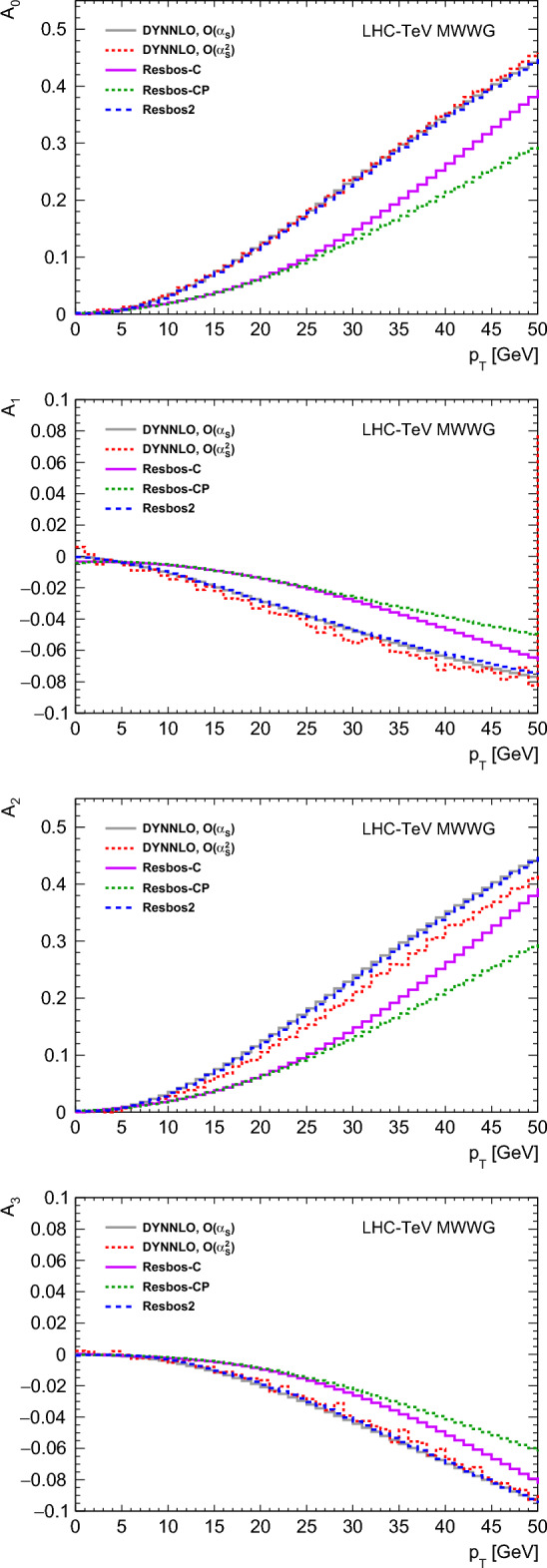
$A_0$ to $A_3$ as a function of $p_{{{\text {T}}}}^{W}$ extracted from ResBos-C, ResBos-CP, ResBos2, DYNNLO at ${{\mathcal {O}}}(\alpha _{{\text {S}}}),$ and DYNNLO at ${{\mathcal {O}}}(\alpha ^2_{{\text {S}}})$ in $p\bar{p}$ collisions at 1.96 TeV. The CTEQ6M PDF set is used for all generators except ResBos-CP, for which CTEQ6.6 is used. The ResBos-C and ResBos2 calculations are at ${{\mathcal {O}}}(\alpha _{\textrm{S}})$ in QCD, and ResBos-CP is at ${{\mathcal {O}}}(\alpha ^2_{{\text {S}}}).$ The difference between DYNNLO at ${{\mathcal {O}}}(\alpha _{{\text {S}}})$ and ${{\mathcal {O}}}(\alpha ^2_{{\text {S}}})$ has an ${{\mathcal {O}}}$(1 MeV) effect on $\delta m_W^{\textrm{pol}}$

The ResBos-C and ResBos-CP codes resum a subset of contributions to Eq. 10, specifically those affecting the $(1 + \cos ^2\theta )$ and $A_4 \cos \theta$ terms. This partial resummation modifies the $A_0$–$A_3$ terms relative to fixed-order predictions, as demonstrated in Fig. 6, where $A_0-A_3$ are shown for *W*-boson events generated at $\sqrt{s}=1.96$ TeV with ResBos-C, ResBos-CP, ResBos2, and DYNNLO. The partial-resummation predictions differ with respect to measurements performed at the LHC [55], which instead agree with fully-resummed calculations such as ResBos2 or Wj-MiNNLO, and fixed-order calculations such as DYNNLO.

Experimental fits for $m_W$ in data use theoretical predictions of the leptonic angular distributions from ResBos-C for CDF, ResBos-CP for D0, DYNNLO [20, 21] for ATLAS, and DYTurbo for LHCb. The CDF experiment applies a post-fit correction to reproduce the NNPDF3.1 PDF prediction, and this correction includes the effect of updating the angular coefficients to those calculated by ResBos2.

**Fig. 7:**
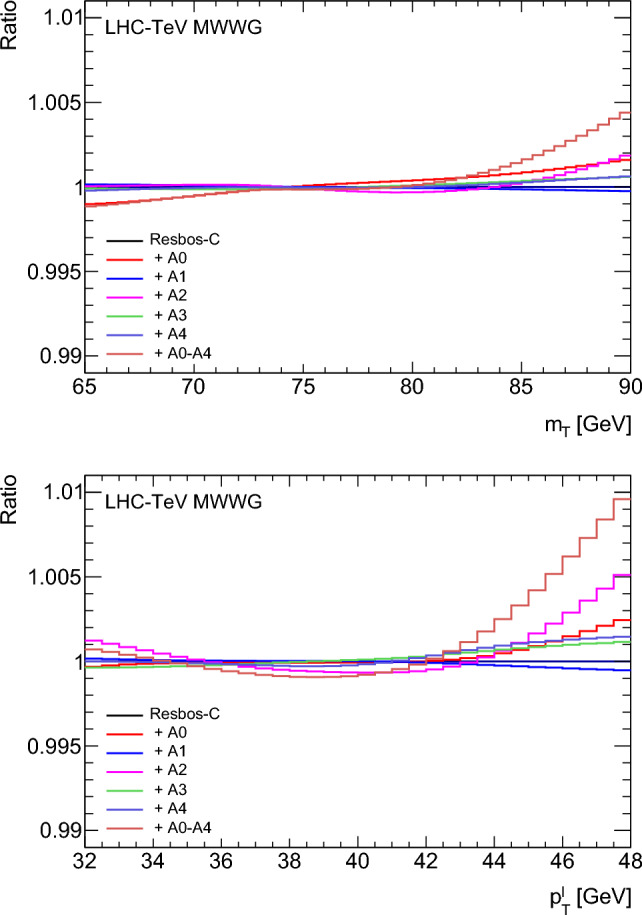
Relative effect of reweighting the $A_0$–$A_4$ coefficients from ResBos-C to ResBos2 on the CDF $m_{{{\text {T}}}}$ (top) and $p_{{{\text {T}}}}^{\ell }$ (bottom) distributions

In order to achieve a common theoretical treatment of the *W*-boson polarization, the results of the CDF and D0 fits to the measurement distributions are adjusted to correspond to the ResBos2 calculation of the leptonic angular distributions at ${{\mathcal {O}}}(\alpha _{\textrm{S}}).$ Events generated with ResBos-C or ResBos-CP are reweighted such that the $A_0-A_4$ coefficients match those of ResBos2, as functions of $p_{{{\text {T}}}}^{W}$ and $y_W.$ The *W*-boson $p_{{{\text {T}}}}$ is fixed to that of the original measurement, in the same manner as for the $\delta m_W^{{\text {PDF}}}$ evaluations in Sect. 4.2.1. The impact of the reweighting on the CDF $m_{{{\text {T}}}}$ and $p_{{{\text {T}}}}^{\ell }$ distributions is shown in Fig. 7, and the $\delta m_W^{\textrm{pol}}$ values from reweighting the $A_i$ coefficients individually and together are given in Tables 7 and 8 for CDF and D0, respectively. The reweighting procedure reproduces the direct fit from ResBos-C or ResBos-CP to ResBos2, as expected since the basis of spherical harmonics is complete and exact. The results of the reweighting procedure for the D0 configuration, $\delta m_W^{\textrm{pol}} = -6.4,$ $-6.9,$ and $-15.8$ MeV for the $m_{{{\text {T}}}}$, $p_{{{\text {T}}}}^{\ell }$, and $p_{{{\text {T}}}}^{\nu }$ distributions, respectively, are applied to the measured $m_W.$ For CDF, values of $\delta m_W^{\textrm{pol}} = -9.5,$ $-8.4,$ and $-12.5$ MeV for the $m_{{{\text {T}}}}$, $p_{{{\text {T}}}}^{\ell }$, and $p_{{{\text {T}}}}^{\nu }$ distributions, respectively, are applied to events generated with ResBos-C.

ATLAS estimates a 5.8 MeV polarization modelling uncertainty based on the precision of measurements on the *Z*-boson resonance, while the LHCb uncertainty of 10 MeV arises from its determination of the $A_3$ coefficient as part of its fit for $m_W.$ These uncertainties are taken to be uncorrelated. The Tevatron experiments do not include a corresponding uncertainty in their measurements. An uncorrelated uncertainty is applied to the shift calculated for each experiment to account for the limitations of the parameterized MWWG simulation. This uncertainty is $\approx 1$ MeV and is similar to that obtained by taking the difference between the NLO and NNLO fixed-order calculations of the leptonic angular coefficients.

### *W*-boson resonance

The details of the event generation for the $m_W$ measurement differ between measurements due to the assumed *W*-boson width ${\Gamma }_W$ and to a restriction on the generated $\ell \nu$ invariant mass range in the CDF sample. These lead to $\delta m_W$ corrections on the direct fits to these samples.

The assumed ${\Gamma }_W$ values used by the experiments are as follows: 2089.5 MeV for the CDF measurement; 2099 and 2100 MeV for the D0 measurements with 1.0 fb$^{-1}$ and 4.3 fb$^{-1},$ respectively; 2094 MeV for the ATLAS measurement; and 2085 for the LHCb measurement. Using the SM prediction of ${\Gamma }_W = 2089.5 \pm 0.6$ MeV leads to $\delta m_W^{\Gamma }=0.0,$ 1.4, 1.5, 0.7, and $-0.7$ MeV for the CDF, D0 1.0 fb$^{-1},$ D0 4.3 fb$^{-1},$ ATLAS, and LHCb measurements, respectively.

The ResBos-C events used in the fit to the CDF data includes an $\ell \nu$ invariant mass requirement of $m_{\ell \nu }<150$ GeV. Differences of up to 10% are observed between Resbos-C and ResBos2 for an invariant mass below 70 GeV, though these have a negligible effect on the measurement. Using the ResBos2 invariant mass distribution without any requirements leads to $\delta m_W^{{\text {gen}}} = -1.6, -3.4,$ and $-3.2$ MeV for the $m_{{{\text {T}}}}$, $p_{{{\text {T}}}}^{\ell }$, and $p_{{{\text {T}}}}^{\nu }$ distributions, respectively, for the CDF fit results. The measured CDF $m_W$ accounts for these effects as part of the update of the PDF set to NNPDF3.1. Smaller differences are observed between Resbos-CP and ResBos2, and there is no significant $\delta m_W^{{\text {gen}}}$ from the invariant mass modelling for the D0 measurement.

**Table 7:** Values of $\delta m_W^{\textrm{pol}}$ in MeV associated with reweighting each $A_i$ coefficient from Resbos-C to Resbos2 for the CDF detector, as well as the result of a direct fit to ResBos2. The result of the direct fit is consistent with that of the reweighting

Coefficient	$m_{{{\text {T}}}}$	$p_{{{\text {T}}}}^{\ell }$	$p_{{{\text {T}}}}^{\nu }$
$A_0$	$-6.3$	$-2.6$	$-9.1$
$A_1$	1.1	1.3	0.3
$A_2$	$-0.7$	0.4	$-3.2$
$A_3$	$-2.1$	$-4.1$	1.0
$A_4$	$-1.4$	$-3.3$	$-1.6$
$A_0-A_4$	$-9.5$	$-8.4$	$-12.5$
ResBos2	$-10.2 \pm 1.1$	$-7.6 \pm 1.2$	$-11.8 \pm 1.4$
Difference	$-0.7 \pm 1.1$	$0.8 \pm 1.2$	$0.7 \pm 1.4$

**Table 8:** Values of $\delta m_W^{\textrm{pol}}$ in MeV associated with reweighting each $A_i$ coefficient from ResBos-CP to Resbos2 for the D0 detector, as well as the result of a direct fit to ResBos2. The result of the direct fit is consistent with that of the reweighting

Coefficient	$m_{{{\text {T}}}}$	$p_{{{\text {T}}}}^{\ell }$	$p_{{{\text {T}}}}^{\nu }$
$A_0$	$-9.8$	$-7.3$	$-15.6$
$A_1$	1.9	2.4	1.8
$A_2$	3.0	3.3	$-2.7$
$A_3$	$-1.6$	$-2.9$	0.4
$A_4$	0.2	$-2.3$	0.5
$A_0-A_4$	$-6.4$	$-6.9$	$-15.8$
ResBos2	$-7.8 \pm 1.0$	$-6.6 \pm 1.1$	$-16.5 \pm 1.2$
Difference	$-1.4 \pm 1.0$	$0.3 \pm 1.1$	$-0.7 \pm 1.2$

### Electroweak corrections

The dominant electroweak effect on the $m_W$ measurement is final-state QED radiation [56], which reduces the momentum of the charged lepton from the *W*-boson decay. The experiments model this radiation using generators that resum multiple soft photon emissions above an energy threshold. Uncertainties on the modelling of electroweak corrections include: (1) the perturbative calculation of photon radiation, including the modelling of single-photon and multi-photon emission and the matching of the fixed-order and all-orders descriptions; (2) the energy threshold for producing final-state photons; and (3) higher-order corrections from final-state $e^+ e^-$ pair production. Tables 9 and 10 list the size of these uncertainties for each experiment in the electron and muon channels, respectively. The uncertainties are completely correlated between the decay channels.

**Table 9:** QED uncertainties in MeV on the $m_W$ measurement in the electron channel using the $m_{{{\text {T}}}}$ ($p_{{{\text {T}}}}$) fit. The uncertainties are uncorrelated except for those due to the perturbative photon radiation calculation, which is taken to be 100% correlated between D0 and ATLAS, and to the photon energy cutoff, taken to be 100% correlated between CDF and D0

Uncertainty	CDF	D0	ATLAS
Perturbative photon rad.	2.3 (2.3)	5 (5)	2.5 (3.3)
Photon energy cutoff	1 (1)	2 (1)	–
FSR $e^+e^-$	1 (1)	–	0.8 (3.6)
Total	2.7 (2.7)	7 (7)	2.6 (4.9)

**Table 10:** QED uncertainties in MeV on the $m_W$ measurement in the muon channel for ATLAS and CDF using the $m_{{{\text {T}}}}$ ($p_{{{\text {T}}}}$) fit, and for LHCb. The uncertainties are taken to be uncorrelated between the experiments

Uncertainty	CDF	ATLAS	LHCb
Perturbative photon rad.	2.3 (2.3)	2.5 (3.5)	8.6
Photon energy cutoff	1 (1)	–	–
FSR $e^+e^-$	1 (1)	0.8 (3.6)	–
Total	2.7 (2.7)	2.6 (5.6)	8.6

To estimate the uncertainty from the limitations of the shower model relative to the matrix-element calculation, D0 and ATLAS perform a direct comparison between PHOTOS and WGRAD [57, 58] or WINHAC [59–61], respectively. Since ATLAS and D0 use the same shower model, their uncertainties are considered as correlated. LHCb estimates the uncertainty with a hybrid approach of comparing Powheg with and without the NLO EW calculation, and the range of the PHOTOS, Pythia8, and Herwig shower models. The average of the measurements from the different shower models is taken as the central value, so the uncertainty is considered as uncorrelated. CDF uses a third strategy, applying a correction to the measurement using the HORACE [62–64] generator, which matches multiple-photon radiation to the $O(\alpha )$ calculation. The residual uncertainties are largely due to MC statistics, and are considered as uncorrelated.

The shower model includes a lower threshold on the emitted photon energy, expressed as a ratio with respect to the energy of the lepton from the *W* boson decay. CDF uses a threshold of $10^{-5}$ and determines the uncertainty by increasing the threshold by a factor of 3. D0 uses a similar procedure except with an increase from $2.5 \times 10^{-4}$ to $2 \times 10^{-2}.$ These uncertainties are taken to be completely correlated.

To account for the higher-order process of an off-shell final-state photon splitting into an $e^+ e^-$ pair, CDF applies an effective radiator approximation to the radiated photons. ATLAS does not apply a correction, instead taking the uncertainty from a PHOTOS model of this process. The uncertainties are treated as uncorrelated.

A higher-order correction not considered by the experiments is the non-factorizable mixed QCD-QED correction, which has not been fully implemented in an event generator. The effect of this correction has been estimated to be of the order of the quoted uncertainties when detector effects are neglected [56], and can increase when different $p_{{{\text {T}}}}^{\ell }$ thresholds are used in *W*- and *Z*-boson selection [65]. These corrections should be considered when they become available.

## Combination

The combination of $m_W$ measurements is performed by first replicating each experiment’s combination of fit results within the experiment, applying any relevant $\delta m_W$ shifts, and then combining across experiments. Section 5.1 describes the procedure to replicate each experiment’s result and to adjust the measurement to a common theoretical model. The adjusted results are compared to the corresponding published values with the same PDF set. The results using a given experiment’s PDF set are compared to the corresponding experimental measurement. Section 5.2 updates all experimental measurements to modern PDF sets and performs the various combinations.

**Table 11:** Published CDF values using the NNPDF3.1 PDF set, and input values to the combination using the results of the direct CDF fits to ResBos-C with the CTEQ6M PDF set. The combination procedure applies shifts to these results to update to the ResBos2 calculation $(\delta m_W^{{\text {pol}}}$ and $\delta m_W^{{\text {gen}}})$ and a shift to update to the NNPDF3.1 PDF set $(\delta m_W^{{\text {PDF}}}).$ The total statistical and systematic uncertainties on the shifts are 1.2, 1.1, and 2.1 MeV for the $m_{{{\text {T}}}}$, $p_{{{\text {T}}}}^{\ell }$, and $p_{{{\text {T}}}}^{\nu }$ fits respectively. The combined value is consistent with that obtained by CDF when using the PDF uncertainties determined by CDF, labelled “Combined $(\sigma _{{\text {PDF}}} = 3.9$ MeV)”. When combining the result with other measurements, the uncertainty is evaluated using NNPDF3.1 eigenvectors to give the result labelled “Combined $(\sigma _{{\text {PDF}}} = 6.6$ MeV)”. The difference is due to a change in the weight of each fit distribution. All units are in MeV

	Published $m_W$	Input $m_W$	$\delta m_W^{{\text {pol}}}$	$\delta m_W^{{\text {gen}}}$	$\delta m_W^{{\text {PDF}}}$	LHC-TeV MWWG $m_W$
	(NNPDF3.1)	(CTEQ6M)				(NNPDF3.1)
$m_{{{\text {T}}}}$ $(e,\nu )$	80,429.1	80,425.8	$-9.5$	$-1.6$	13.1	80,427.8
$p_{{{\text {T}}}}^{\ell }$(*e*)	80,411.4	80,407.8	$-8.4$	$-3.4$	14.0	80,410.0
$p_{{{\text {T}}}}^{\nu }$(*e*)	80,426.3	80,423.3	$-12.5$	$-3.2$	15.1	80,422.7
$m_{{{\text {T}}}}$ $(\mu ,\nu )$	80,446.1	80,442.8	$-9.5$	$-1.6$	13.1	80,444.8
$p_{{{\text {T}}}}^{\ell }$ $(\mu )$	80,428.2	80,424.6	$-8.4$	$-3.4$	14.0	80,426.8
$p_{{{\text {T}}}}^{\nu }$ $(\mu )$	80,428.9	80,425.9	$-12.5$	$-3.2$	15.1	80,425.3
Combined $(\sigma _{{\text {PDF}}} = 3.9$ MeV)	80,433.5					80,432.1
Combined $(\sigma _{{\text {PDF}}} = 6.6$ MeV)						80,433.3

**Table 12:** Published D0 values corresponding to the CTEQ6.1 (Run 2a) and CTEQ6.6 (Run 2b) PDF sets, along with the following shifts: modifying the leptonic angular distributions to match those of ResBos2 $(\delta m_W^{{\text {pol}}});$ modifying the Run 2a result to correspond to the CTEQ6.6 PDF set $(\delta m_W^{{\text {PDF}}});$ and modifying the *W* boson width to the Standard Model prediction $(\delta m_W^{\Gamma }).$ The total statistical and systematic uncertainties on the shifts are 1.2, 1.2, and 2.3 MeV for the $m_{{{\text {T}}}}$, $p_{{{\text {T}}}}^{\ell }$, and $p_{{{\text {T}}}}^{\nu }$ fits respectively. The combined result with the published D0 PDF uncertainty obtained using Pythia and the CTEQ6.1 PDF set is labelled “Combined $(\sigma _{{\text {PDF}}} = 11$ MeV)”, and the result with PDF uncertainties updated to those of CTEQ6.6 calculated with Wj-MiNNLO is labelled “Combined $(\sigma _{{\text {PDF}}} = 15.1$ MeV)”. The results differ due to different weights of the individual fits to kinematic distributions. All units are in MeV

	Published $m_W$	$\delta m_W^{{\text {pol}}}$	$\delta m_W^{{\text {PDF}}}$	$\delta m_W^{\Gamma }$	LHC-TeV MWWG $m_W$
	(CTEQ6.1, CTEQ6.6)				(CTEQ6.6)
Run 2a $m_{{{\text {T}}}}$ $(e,\nu )$	80,401	$-6.4$	14.3	1.4	80,410.3
Run 2a $p_{{{\text {T}}}}^{\ell }$(*e*)	80,400	$-6.9$	16.7	1.4	80,411.2
Run 2a $p_{{{\text {T}}}}^{\nu }$(*e*)	80,402	$-15.8$	17.5	1.4	80,405.1
Run 2b $m_{{{\text {T}}}}$ $(e,\nu )$	80,371	$-6.4$		1.5	80,366.1
Run 2b $p_{{{\text {T}}}}^{\ell }$(*e*)	80,343	$-6.9$		1.5	80,337.6
Combined $(\sigma _{{\text {PDF}}} = 11$ MeV)	80,375				80,373.4
Combined $(\sigma _{{\text {PDF}}} = 15.1$ MeV)					80,377.9

### Procedures

The CDF individual $m_W$ values using the $m_{{{\text {T}}}}$, $p_{{{\text {T}}}}^{\ell }$, and $p_{{{\text {T}}}}^{\nu }$ distributions in the electron and muon channels are combined using the reported uncertainties and correlations, giving the results shown in Table 11. The CDF measurement applies $\delta m_W$ values of 3.3,  3.6,  and 3.0 MeV, respectively, to fits to ResBos-C with the CTEQ6M PDF set. We remove these $\delta m_W$ corrections and add $\delta m_W^{{\text {pol}}} + \delta m_W^{{\text {gen}}} = -11.1,$ $-11.8,$ and $-15.7$ MeV to the $m_{{{\text {T}}}}$, $p_{{{\text {T}}}}^{\ell }$, and $p_{{{\text {T}}}}^{\nu }$ results, respectively, corresponding to the ResBos2 calculation of leptonic angular distributions described in Sect. 4.3 and the removal of the $\ell \nu$ invariant mass requirement discussed in Sect. 4.4. Finally, a shift to the target PDF set is applied. For the NNPDF3.1 PDF set this procedure gives a combined CDF value of $m_W = 80432.1 \pm 9.4$ MeV, which is consistent with the published CDF value of $m_W = 80433.5 \pm 9.4$ MeV within the uncertainty of the procedure. Given this agreement it is apparent that the $\delta m_W$ shifts applied in the CDF analysis include both the PDF shift from CTEQ6M to NNPDF3.1 and the polarization and generator shifts from ResBos-C to ResBos2.

**Table 13:** The CDF and D0 Run 2 $m_W$ and $\chi ^2$ values obtained from a combination of the individual measurement distributions and decay channels, along with the combined Tevatron Run 2 $m_W,$ PDF uncertainty, $\chi ^2,$ and probability of obtaining this $\chi ^2$ or larger. Mass units are in MeV

PDF set	CDF (5 d.o.f.)		D0 (4 d.o.f.)		Tevatron Run 2 (1 d.o.f.)			
	$m_W$	$\chi ^2$	$m_W$	$\chi ^2$	$m_W$	$\sigma _{{\text {PDF}}}$	$\chi ^2$	p$(\chi ^2,n)$
ABMP16	$80{,}417.3~\pm ~9.5$	8.8	$80{,}355.4 \pm 20.9$	6.6	$80{,}408.2 \pm 8.9$	4.0	7.7	0.6%
CT14	$80{,}432.1 \pm 15.5$	7.7	$80{,}370.9 \pm 24.9$	5.9	$80{,}424.0 \pm 15.2$	12.6	7.2	0.7%
CT18	$80{,}432.0 \pm 16.1$	7.6	$80{,}372.0 \pm 25.5$	5.9	$80{,}424.9 \pm 15.9$	13.5	7.0	0.8%
MMHT2014	$80{,}425.7 \pm 11.6$	7.0	$80{,}364.4 \pm 22.3$	5.5	$80{,}417.4 \pm 11.2$	7.8	7.6	0.6%
MSHT20	$80{,}424.4 \pm 12.2$	7.6	$80{,}362.3 \pm 22.5$	6.1	$80{,}415.9 \pm 11.8$	8.6	7.8	0.5%
NNPDF3.1	$80{,}433.3 \pm 10.9$	7.6	$80{,}372.7 \pm 21.9$	5.8	$80{,}425.0 \pm 10.5$	6.8	7.4	0.7%
NNPDF4.0	$80{,}441.8 \pm 11.6$	7.2	$80{,}381.3 \pm 22.2$	5.7	$80{,}433.4 \pm 11.2$	7.8	7.4	0.7%

**Table 14:** The ATLAS and LHCb $m_W$ values obtained from a combination of the individual measurement distributions and decay channels, along with the combined LHC $m_W,$ PDF uncertainty, and $\chi ^2,$ and probability of obtaining this $\chi ^2$ or larger. The $\chi ^2$ of the combination of fit distributions and decay channels is shown for ATLAS; no $\chi ^2$ is shown for LHCb as the measurement is performed using one distribution in one channel. Mass units are in MeV

PDF set	ATLAS (27 d.o.f)		LHCb		LHC (1 d.o.f)			
	$m_W$	$\chi ^2$	$m_W$	$\chi ^2$	$m_W$	$\sigma _{{\text {PDF}}}$	$\chi ^2$	p$(\chi ^2,n)$
ABMP16	$80{,}352.8 \pm 16.1$	31	$80{,}361.0 \pm 30.4$	–	$80{,}354.6 \pm 14.2$	2.9	0.1	75%
CT14	$80{,}363.1 \pm 20.4$	30	$80{,}354.4 \pm 32.2$	–	$80{,}360.4 \pm 16.4$	6.5	0.0	100%
CT18	$80{,}374.5 \pm 20.3$	30	$80{,}347.3 \pm 32.7$	–	$80{,}366.5 \pm 16.6$	6.3	0.5	48%
MMHT2014	$80{,}372.8 \pm 18.6$	30	$80{,}342.5 \pm 31.3$	–	$80{,}364.4 \pm 15.4$	5.1	0.6	44%
MSHT20	$80{,}368.9 \pm 17.9$	45	$80{,}351.3 \pm 31.0$	–	$80{,}364.3 \pm 15.0$	4.5	0.2	65%
NNPDF3.1	$80{,}358.4 \pm 17.6$	29	$80{,}359.3 \pm 31.1$	–	$80{,}358.6 \pm 15.0$	5.0	0.0	100%
NNPDF4.0	$80{,}353.5 \pm 16.6$	35	$80{,}361.6 \pm 30.6$	–	$80{,}355.4 \pm 14.5$	3.8	0.1	75%

The principal component analysis used by CDF to reduce statistical effects in the PDF uncertainty evaluation is not used in the combination with other experiments, since different measurements would give different principal components and complicate the correlation evaluations for the combination. Instead the Hessian sets provided by the NNPDF collaboration are used to estimate the PDF uncertainty. The combined CDF value with this uncertainty is labelled “Combined $(\sigma _{{\text {PDF}}} = 6.6$ MeV)” in Table 11 and corresponds to the entry labelled “NNPDF 3.1” in Table 13 in Sect. 5.2.

The individual D0 measurements with the $m_{{{\text {T}}}}$ and $p_{{{\text {T}}}}^{\ell }$ distributions using data sets corresponding to 1.1 fb$^{-1}$ and 4.3 fb$^{-1}$ are combined using the reported uncertainties to give the result $m_W = 80375.1 \pm 23.1$ MeV, which is the value quoted by D0 rounded to the nearest MeV. Before combining with other measurements, a number of shifts are applied. First, a shift from CTEQ6.1 to CTEQ6.6 is applied to the measurement based on 1.1 fb$^{-1}$ of integrated luminosity. Shifts of $\delta m_W^{{\text {pol}}} = -6.4,$ $-6.9,$ and $-15.8$ are applied to the $m_{{{\text {T}}}}$, $p_{{{\text {T}}}}^{\ell }$, and $p_{{{\text {T}}}}^{\nu }$ fit results, respectively, to update the ResBos-CP leptonic angular distributions to those of ResBos2. Finally, a $\delta m_W^{\Gamma }$ shift adjusts ${\Gamma _W}$ to that of the SM prediction. The result with these shifts and the published D0 PDF uncertainty of $\approx 11$ MeV is labelled “Combined $(\sigma _{{\text {PDF}}} = 11$ MeV)” in Table 12. The value with uncertainties updated to those calculated with Wj-MiNNLO and CTEQ6.6 is $m_W = 80377.9 \pm 25.5$ MeV and is labelled “Combined $(\sigma _{{\text {PDF}}} = 15.1$ MeV)” in the table.

The ATLAS measurement is reproduced using the parameterized simulation to give a value of $m_W = 80{,}369.7 \pm 18.5$ MeV, which is within a few tenths of an MeV of the published result. A $\delta m_W^{\Gamma } = 0.7$ MeV correction is added to update ${\Gamma }_W,$ and further $\delta m_W^{{\text {PDF}}}$ shifts are applied to provide the central value for the target PDF set. All LHCb $m_W$ values for the combination are determined from a direct fit to the data using the target PDF set, so only a $\delta m_W^{\Gamma } = -0.7$ MeV shift is applied to update the value of the *W* boson width.

### Results

A series of combinations are performed corresponding to the Tevatron Run 2 experiments, the LHC experiments, all experiments including the result from the LEP combination, and all experiments except one. For each experiment the central value, uncertainty, and $\chi ^2$ of the individual measurements is shown for the ABMP16, CT14, CT18, MMHT2014, MSHT20, NNPDF31, and NNPDF40 PDF sets. For the combined result of multiple experiments the overall PDF uncertainty is also shown. The PDF uncertainties for the individual experiments are given in Table 4.

#### Hadron-collider measurements

Results for the Tevatron Run 2 experiments are listed in Table 13. The individual combinations of the CDF and D0 fit results are satisfactory for all PDF sets, with probabilities ranging from 12 to 24%. The Tevatron-wide combination has a total uncertainty ranging from 8.9 MeV for ABMP16 to 15.9 MeV for CT18, and a $\chi ^2$ probability of 0.5–0.8%.

As discussed in Sect. 4.2.1, PDF uncertainties are fully correlated between CDF and D0. The PDF uncertainty in the combination is therefore close to that obtained for each experiment, and ranges from 4 MeV for ABMP16 to 13.5 MeV for CT18. The combined central value ranges from 80,408.2 MeV for ABMP16 to 80,433.4 MeV for NNPDF4.0. The difference between the NNPDF3.1 and NNPDF4.0 combinations, 8.4 MeV, is similar to the PDF uncertainty of the NNPDF4.0 set (7.8 MeV). Similar trends are observed for the CDF and D0 measurements separately. Some variation in the results with PDF set is expected due to differences in input data sets to the PDFs, and to the differences in the compatibility with Drell–Yan measurements discussed in Sect. 4.2.2. Further understanding of these differences would benefit future combinations.

The LHC results are summarized in Table 14. The $\chi ^2$ per degree of freedom of the ATLAS combination ranges from 29/27 (for NNPDF3.1) to 45/27 (for MSHT20). The latter corresponds to a probability of about 2%. The larger $\chi ^2$ for MSHT20 is consistent with the calculations of Drell–Yan measurements. The ATLAS and LHCb measurements are compatible and have a total uncertainty ranging from 14.2 to 16.6 MeV.

**Fig. 8:**
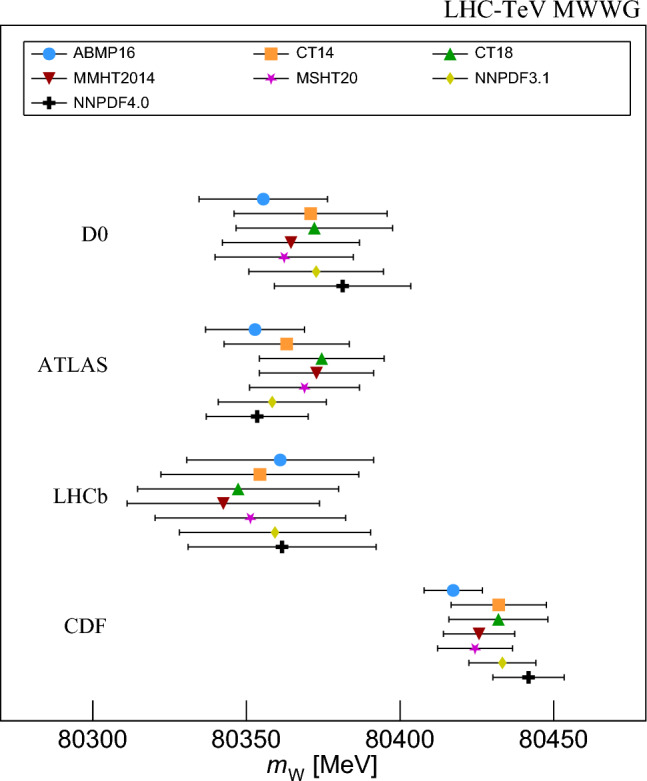
The D0, ATLAS, LHCb, and CDF $m_W$ values and uncertainties using the ABMP16, CT14, CT18, MMHT2014, MSHT20, NNPDF3.1, and NNPDF4.0 PDF sets

The individual experimental results are shown in Fig. 8 for all considered PDF sets. The combination of ATLAS and LHCb measurements benefits from anti-correlated PDF uncertainties [66]. Therefore the combined PDF uncertainties and the variation of the combined central values are smaller than for the individual experiments. The ATLAS $m_W$ value ranges from 80,352.8 MeV for ABMP16 to 80,374.5 MeV for CT18. This range is comparable to that of the Tevatron experiments. A similar spread but opposite trends are observed for LHCb, and the spread of $m_W$ values is reduced from $\approx 20$ MeV to 14.1 MeV in the combination. The PDF uncertainties range from 4.0 to 11.4 MeV for ATLAS and 3.0 to 12.2 MeV for LHCb, but are reduced to 2.9–6.5 MeV for the combined result.

**Table 15:** Combination of $m_W$ measurements from the individual experiments. Shown for each PDF are the PDF uncertainty, $\chi ^2,$ and probability of obtaining this $\chi ^2$ or larger. Mass units are in MeV

All experiments (4 d.o.f.)				
PDF set	$m_W$	$\sigma _{{\text {PDF}}}$	$\chi ^2$	p$(\chi ^2,n)$
ABMP16	$80{,}392.7 \pm 7.5$	3.2	29	0.0008%
CT14	$80{,}393.0 \pm 10.9$	7.1	16	0.3%
CT18	$80{,}394.6 \pm 11.5$	7.7	15	0.5%
MMHT2014	$80{,}398.0 \pm 9.2$	5.8	17	0.2%
MSHT20	$80{,}395.1 \pm 9.3$	5.8	16	0.3%
NNPDF3.1	$80{,}403.0 \pm 8.7$	5.3	23	0.1%
NNPDF4.0	$80{,}403.1 \pm 8.9$	5.3	28	0.001%

**Table 16:** Combination of all $m_W$ measurements except the LEP average. Shown for each PDF are the PDF uncertainty, $\chi ^2,$ and probability of obtaining this $\chi ^2$ or larger. Mass units are in MeV

All except LEP (3 d.o.f.)				
PDF set	$m_W$	$\sigma _{{\text {PDF}}}$	$\chi ^2$	p$(\chi ^2,n)$
ABMP16	$80{,}393.6 \pm 7.8$	3.4	19	0.03%
CT14	$80{,}395.1 \pm 11.6$	8.0	16	0.1%
CT18	$80{,}397.1 \pm 12.3$	8.8	15	0.2%
MMHT2014	$80{,}399.9 \pm 9.6$	6.2	17	0.7%
MSHT20	$80{,}396.8 \pm 9.7$	6.3	16	0.1%
NNPDF3.1	$80{,}405.0 \pm 9.0$	5.6	22	0.007%
NNPDF4.0	$80{,}405.3 \pm 9.2$	5.7	27	0.0006%

**Table 17:** Combination of $m_W$ measurements from all individual experiments except CDF. Shown for each PDF are the PDF uncertainty, $\chi ^2,$ and probability of obtaining this $\chi ^2$ or larger. Mass units are in MeV

All except CDF (3 d.o.f.)				
PDF set	$m_W$	$\sigma _{{\text {PDF}}}$	$\chi ^2$	p$(\chi ^2,n)$
ABMP16	$80{,}357.3 \pm 11.2$	2.6	0.4	94%
CT14	$80{,}365.4 \pm 12.9$	5.8	0.3	96%
CT18	$80{,}369.2 \pm 13.3$	6.2	0.5	92%
MMHT2014	$80{,}365.8 \pm 12.1$	4.7	0.8	85%
MSHT20	$80{,}365.1 \pm 12.0$	4.4	0.4	94%
NNPDF3.1	$80{,}364.7 \pm 11.9$	4.5	0.4	94%
NNPDF4.0	$80{,}364.5 \pm 11.6$	3.9	1.2	75%

**Table 18:** Combination of $m_W$ measurements from all individual experiments except D0. Shown for each PDF are the PDF uncertainty, $\chi ^2,$ and probability of obtaining this $\chi ^2$ or larger. Mass units are in MeV

All except D0 (3 d.o.f.)				
PDF set	$m_W$	$\sigma _{{\text {PDF}}}$	$\chi ^2$	p$(\chi ^2,n)$
ABMP16	$80{,}397.2 \pm 7.8$	3.1	15	0.2%
CT14	$80{,}395.9 \pm 11.0$	7.0	11	1.2%
CT18	$80{,}397.1 \pm 11.6$	7.6	10	1.9%
MMHT2014	$80{,}401.9 \pm 9.4$	5.6	13	0.5%
MSHT20	$80{,}399.0 \pm 9.5$	5.7	12	0.7%
NNPDF3.1	$80{,}406.6 \pm 8.9$	5.1	19	0.03%
NNPDF4.0	$80{,}406.4 \pm 9.0$	5.1	25	0.002%

**Table 19:** Combination of $m_W$ measurements from the individual experiments except for ATLAS. Shown for each PDF are the PDF uncertainty, $\chi ^2,$ and probability of obtaining this $\chi ^2$ or larger. Mass units are in MeV

All except ATLAS (3 d.o.f.)				
PDF set	$m_W$	$\sigma _{{\text {PDF}}}$	$\chi ^2$	p$(\chi ^2,n)$
ABMP16	$80{,}402.8 \pm 8.3$	3.5	11	1.2%
CT14	$80{,}406.5 \pm 12.8$	9.1	12	0.7%
CT18	$80{,}405.0 \pm 13.2$	9.6	13	0.5%
MMHT2014	$80{,}405.8 \pm 10.0$	6.3	14	0.3%
MSHT20	$80{,}404.2 \pm 10.3$	6.6	12	0.7%
NNPDF3.1	$80{,}414.7 \pm 9.5$	5.6	13	0.5%
NNPDF4.0	$80{,}420.2 \pm 10.0$	6.2	14	0.3%

**Table 20:** Combination of $m_W$ measurements from the individual experiments except for LHCb. Shown for each PDF are the PDF uncertainty, $\chi ^2,$ and probability of obtaining this $\chi ^2$ or larger. Mass units are in MeV

All except LHCb (3 d.o.f.)				
PDF set	$m_W$	$\sigma _{{\text {PDF}}}$	$\chi ^2$	p$(\chi ^2,n)$
ABMP16	$80{,}394.8 \pm 7.7$	3.4	18	0.04%
CT14	$80{,}399.8 \pm 11.7$	8.4	14	0.3%
CT18	$80{,}402.6 \pm 12.4$	9.0	12	0.7%
MMHT2014	$80{,}404.4 \pm 9.7$	6.5	13	0.5%
MSHT20	$80{,}400.7 \pm 10.0$	6.8	14	0.3%
NNPDF3.1	$80{,}407.4 \pm 9.1$	5.8	20	0.02%
NNPDF4.0	$80{,}407.3 \pm 9.3$	5.9	26	0.001%

#### All measurements

Tables 15, 16, 17, 18, 19 and 20 provide the results for various combinations including LEP, whose uncertainties are treated as uncorrelated with the others. A combination of all measurements yields a total uncertainty ranging between 7.5 and 11.5 MeV, though the $\chi ^2$ probabilities are low, ranging from $8 \times 10^{-6}$ to $5\times 10^{-3}.$ The low probabilities reflect the discrepancy between the CDF measurement and the other measurements. The combined value of $m_W$ for the CT18 PDF set, which gives the largest compatibility with the broader Drell–Yan measurements, is $m_W = 80{,}394.6 \pm 11.5$ MeV with a probability of 0.5%. The relative weights of the CDF, ATLAS, LHCb, LEP, and D0 measurements are 41%, 28%, 13%, 12%, and 5%, respectively. Weights for other PDF sets are given in the Appendix. The largest difference in $m_W$ between PDF sets is 10.4 MeV.

A possible procedure for combining measurements with low compatibility is to scale all uncertainties by the square root of the ratio of the $\chi ^2$ to the number of degrees of freedom. This procedure effectively assumes a common underestimated uncertainty, which is an unlikely scenario for these measurements. The PDF uncertainty is only partially correlated, and the uncertainty from the CT18 set is the most conservative. Other measurement uncertainties are smaller or are statistically constrained and therefore uncorrelated.

To evaluate the significance of differences between individual measurements and the others, separate combinations are performed excluding, in turn, each individual result from the average. Removing LEP, D0, or LHCb from the combination increases the uncertainty by up to 0.9 MeV and affects the central value by up to 8 MeV. When removing ATLAS the $\chi ^2$ probability ranges from 0.3 to 1.2%, and the uncertainty ranges from 8.3 to 13.2 MeV. The combinations with CDF excluded have good compatibility and the total uncertainty increases to 11.2–13.3 MeV, or 2–4 MeV more than the full combination. The variation of this combination with PDF set is 11.9 MeV, with the value for the ABMP16 PDF set considerably lower than the others (the variation is 4.5 MeV without this set). The combination of all measurements except CDF is $m_W=80{,}369.2 \pm 13.3$ MeV for the CT18 PDF set, with a 91% probability for the $\chi ^2$ to be larger than that observed. The relative weights for the ATLAS, D0, LHCb, and LEP measurements are 42%, 23%, 18%, and 16%, respectively.

**Fig. 9:**
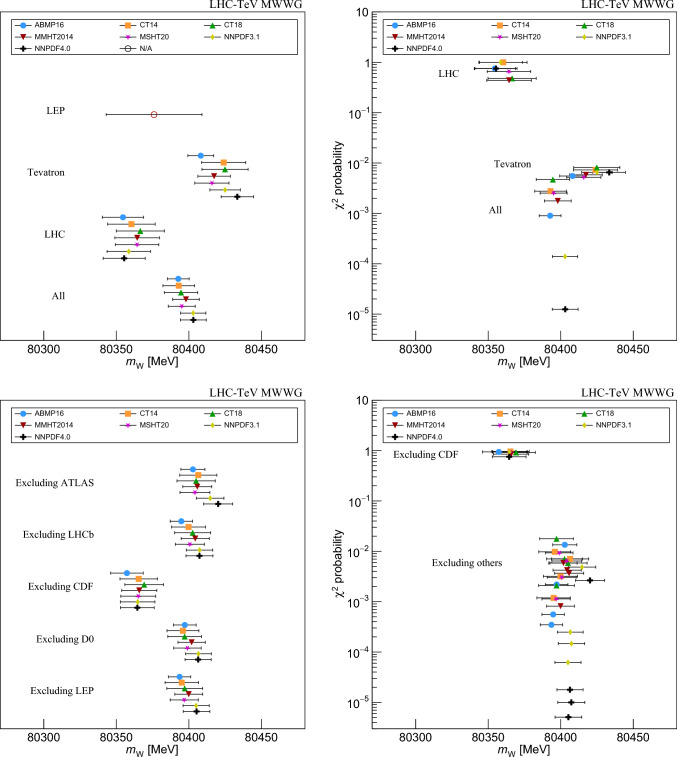
Top left: The combined $m_W$ values and uncertainties from LEP, the Tevatron, LHC, and all experiments, using the ABMP16, CT14, CT18, MMHT2014, MMHT20, NNPDF3.1, and NNPDF4.0 PDF sets. Top right: The corresponding probability of consistency determined using the $\chi ^2$ per degrees of freedom. Bottom left: The combined $m_W$ values and uncertainties for all experiments except one using the ABMP16, CT14, CT18, MMHT2014, MMHT20, NNPDF3.1, and NNPDF4.0 PDF sets. Bottom right: The corresponding probability of consistency determined using the $\chi ^2$ per degrees of freedom

The partial combinations are also used to evaluate the difference between each experimental result and the combination of the others. Considering all PDF sets, the LEP result is compatible with the average of the others to better than one standard deviation. The compatibility of D0 or LHCb with the rest ranges from 1–1.8 standard deviations. The ATLAS result differs from the others by 1.6–3.6 standard deviations, where the largest difference is obtained with the NNPDF4.0 PDF set. Finally, the CDF measurement differs from the others by 3.6–5.2 standard deviations, depending on the choice of the PDF set. The smallest significance corresponds to the CT18 set and the largest significance corresponds to the NNPDF4.0 set.

The $m_W$ combinations from LEP, the Tevatron, the LHC, and all experiments are presented in Fig. 9 for all PDF sets, along with the corresponding $\chi ^2$ probabilities. The same information is also shown for the combinations removing one experimental result at a time.

## Conclusion

A combination of $m_W$ measurements from the CDF, D0, ATLAS, LHCb, and combined LEP experiments has been performed. Where necessary, measurement results have been updated to incorporate an improved theoretical description of the final state distributions. Experimental resolution effects, which are required to propagate the impact of variations in the theoretical description of *W*-boson production and decay, are accounted for using a realistic emulation of the ATLAS, CDF, and D0 measurement procedures. Results for LHCb are produced using the published analysis procedures.

The largest theoretical uncertainty arises from the parton distribution functions. Results are presented for the two most recent PDF sets from the NNPDF, CTEQ, and M(M/S)HT collaborations, as well as the most recent set from the ABMP collaboration. Partial or negative correlations of PDF uncertainties between the Tevatron, ATLAS, and LHCb experiments reduce the dependence of the combined result on the PDF set. This dependence is nonetheless significant, as the differences between individual sets is of the same order as the associated uncertainty. The dependence of the measurements on PDF set are due to differences in the input data sets and to the modelling assumptions in the PDFs, and could ultimately limit the precision of future $m_W$ measurements and combinations at hadron colliders. Improving the experimental precision on $m_W$ requires a better understanding of PDF model dependence, and of uncertainty correlations between PDF sets.

The consistency of Drell–Yan cross-section measurements, as well as the $m_W$ combination, is highest for the CT18 PDF set due to its large uncertainties. With this PDF set the combination of LEP, LHC, and Tevatron Run 2 measurements gives a value $m_W = 80{,}394.6 \pm 11.5$ MeV. This value has a $\chi ^2$ probability of $0.5\%$ and is therefore disfavoured. Other PDF sets give probabilities of consistency between $2\times 10^{-5}$ and $3\times 10^{-3}.$

Good consistency is observed when all experiments other than CDF are combined, with a resulting *W*-boson mass of $80{,}369.2 \pm 13.3$ MeV and a 91% probability of consistency for the CT18 PDF set. When using this set and uncertainty for the CDF measurement and for the combination of the others, the values differ by 3.6 standard deviations. Further measurements or studies of procedures and uncertainties are required to improve the understanding and consistency of a world-average value of the *W* boson mass.

**Fig. 10:**
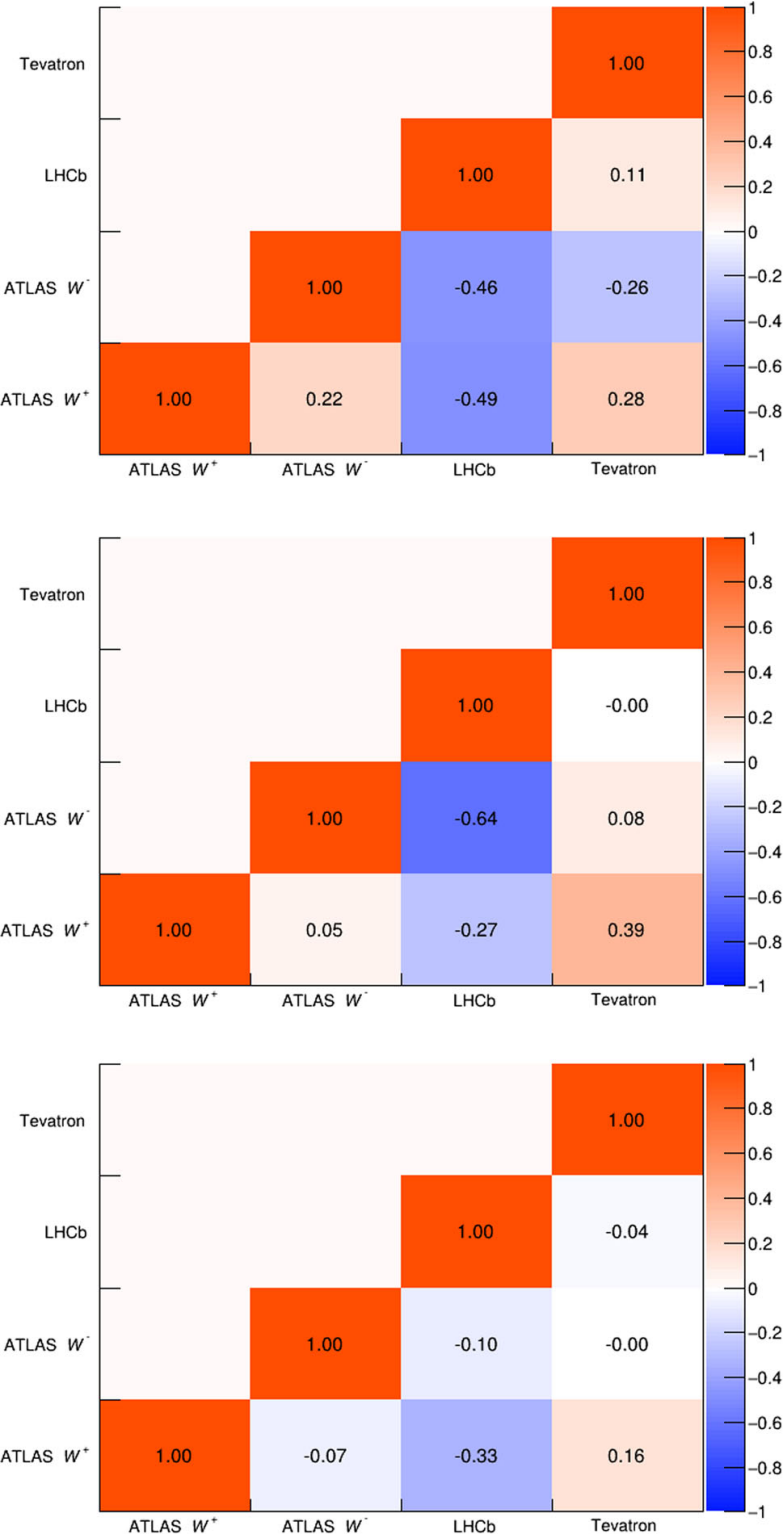
PDF uncertainty correlation matrices for the CT14, MMHT2014, and NNPDF3.1 PDF sets, shown from top to bottom

## Data Availability

This manuscript has no associated data or the data will not be deposited. [Authors’ comment: The Monte Carlo data used for this analysis can be made available through the working group contacts. For further information see the working group webpage, https://twiki.cern.ch/twiki/bin/view/LHCPhysics/LHC-TEV-MWWG.]

## Appendix A: Further information

We provide here additional information on the combination inputs and results. Figure 10 shows the correlation matrices for the hadron-collider measurements for the CT14, MMHT2014, NNPDF3.1 PDF sets. Tables 21, 22 and 23 give the relative weight of each measurement to various combinations of measurements (Tables 24, 25, 26, 27, 28).

**Table 21:** Relative weights (in percent) of the CDF and D0 measurements for the Tevatron combination

Measurement	ABMP16	CT14	CT18	MMHT2014	MSHT20	NNPDF3.1	NNPDF4.0
CDF	85.3	86.8	88.1	86.4	86.3	86.2	86.1
D0	14.7	13.2	11.9	13.6	13.7	13.8	13.9

**Table 22:** Relative weights (in percent) of the ATLAS and LHCb measurements for the LHC combination

Measurement	ABMP16	CT14	CT18	MMHT2014	MSHT20	NNPDF3.1	NNPDF4.0
ATLAS	77.8	69.3	70.5	72.4	73.7	74.9	77.0
LHCb	22.2	30.7	29.5	27.6	26.3	25.1	23.0

**Table 23:** Relative weights (in percent) of individual measurements for the combination of all available measurements

Measurement	ABMP16	CT14	CT18	MMHT2014	MSHT20	NNPDF3.1	NNPDF4.0
ATLAS	19.9	28.2	28.2	20.2	22.8	19.7	24.8
LHCb	6.0	12.7	13.4	9.7	10.7	8.5	8.9
CDF	58.8	42.2	41.1	54.0	50.6	56.1	51.0
D0	10.1	6.0	5.1	8.3	7.9	8.7	8.1
LEP	5.2	10.9	12.2	7.7	8.0	7.0	7.2

**Table 24:** Relative weights (in percent) of individual measurements for the combination of all except the LEP measurement

Measurement	ABMP16	CT14	CT18	MMHT2014	MSHT20	NNPDF3.1	NNPDF4.0
ATLAS	21.0	31.6	32.1	21.9	24.7	21.2	26.7
LHCb	6.3	14.3	15.3	10.5	11.7	9.1	9.5
CDF	62.1	47.3	46.8	58.6	55.0	60.4	55.0
D0	10.6	6.7	5.8	9.0	8.6	9.3	8.7

**Table 25:** Relative weights (in percent) of individual measurements for the combination of all except the CDF measurement

Measurement	ABMP16	CT14	CT18	MMHT2014	MSHT20	NNPDF3.1	NNPDF4.0
ATLAS	47.4	41.5	42.2	42.5	44.2	44.4	47.5
LHCb	13.7	18.6	18.4	17.1	17.0	15.8	14.9
D0	27.5	24.6	23.1	26.9	25.7	26.8	25.3
LEP	11.5	15.3	16.2	13.5	13.1	13.0	12.4

**Table 26:** Relative weights (in percent) of individual measurements for the combination of all except the D0 measurement

Measurement	ABMP16	CT14	CT18	MMHT2014	MSHT20	NNPDF31	NNPDF40
ATLAS	21.9	28.8	28.7	21.5	24.0	21.3	26.2
LHCb	6.5	13.0	13.6	10.1	11.1	8.9	9.2
CDF	66.0	47.2	45.4	60.4	56.7	62.5	57.1
LEP	5.6	11.1	12.3	8.0	8.2	7.3	7.5

**Table 27:** Relative weights (in percent) of individual measurements for the combination of all except the ATLAS measurement

Measurement	ABMP16	CT14	CT18	MMHT2014	MSHT20	NNPDF3.1	NNPDF4.0
LHCb	7.2	14.8	15.7	10.4	12.1	9.4	11.0
CDF	73.8	61.0	60.4	69.5	67.4	71.0	68.7
D0	12.7	9.2	7.9	10.9	10.7	11.3	11.1
LEP	6.3	15.0	16.0	9.2	9.8	8.3	9.2

**Table 28:** Relative weights (in percent) of individual measurements for the combination of all except the LHCb measurement

Measurement	ABMP16	CT14	CT18	MMHT2014	MSHT20	NNPDF3.1	NNPDF4.0
ATLAS	21.0	30.1	30.7	21.1	24.6	21.0	27.1
CDF	62.7	50.0	48.8	60.8	57.3	61.7	56.1
D0	10.8	7.2	6.2	9.4	9.0	9.6	8.9
LEP	5.5	12.7	14.2	8.7	9.1	7.7	8.0

## Footnotes

1 We use the convention $c \equiv 1.$

2 We work in a right-handed coordinate system with the origin at the nominal centre of the detector and the *z*-axis along the nominal direction of the beams. Spherical coordinates $(r, \phi , \theta )$ are also used, with $\phi$ the azimuthal angle around the *z*-axis. The pseudorapidity is defined in terms of the polar angle $\theta$ as $\eta = -\ln \tan (\theta /2).$ Momentum in the plane transverse to the *z*-axis is defined as $p_{{{\text {T}}}}= p\sin \theta .$

## Code availability

My manuscript has no associated code/software. [Author’s comment: The code used to simulate detector response is available in a git repository. For further information see the working group webpage, https://twiki.cern.ch/twiki/bin/view/LHCPhysics/LHC-TEV-MWWG.]
